# Single-cell transcriptomics reveals the brain evolution of web-building spiders

**DOI:** 10.1038/s41559-023-02238-y

**Published:** 2023-11-02

**Authors:** Pengyu Jin, Bingyue Zhu, Yinjun Jia, Yiming Zhang, Wei Wang, Yunxiao Shen, Yu Zhong, Yami Zheng, Yang Wang, Yan Tong, Wei Zhang, Shuqiang Li

**Affiliations:** 1 Key Laboratory of Zoological Systematics and Evolution, Institute of Zoology, Chinese Academy of Sciences, Beijing, China; 2 University of Chinese Academy of Sciences, Beijing, China; 3 School of Life Sciences, IDG/McGovern Institute for Brain Research, Tsinghua University, Beijing, China; 4 Tsinghua-Peking Center for Life Sciences, Beijing, China; 5 Guangxi Normal University, Guilin, China

**Keywords:** Evolution, Zoology

## Abstract

Spiders are renowned for their efficient capture of flying insects using intricate aerial webs. How the spider nervous systems evolved to cope with this specialized hunting strategy and various environmental clues in an aerial space remains unknown. Here we report a brain-cell atlas of >30,000 single-cell transcriptomes from a web-building spider (*Hylyphantes graminicola*). Our analysis revealed the preservation of ancestral neuron types in spiders, including the potential coexistence of noradrenergic and octopaminergic neurons, and many peptidergic neuronal types that are lost in insects. By comparing the genome of two newly sequenced plesiomorphic burrowing spiders with three aerial web-building spiders, we found that the positively selected genes in the ancestral branch of web-building spiders were preferentially expressed (42%) in the brain, especially in the three mushroom body-like neuronal types. By gene enrichment analysis and RNAi experiments, these genes were suggested to be involved in the learning and memory pathway and may influence the spiders’ web-building and hunting behaviour. Our results provide key sources for understanding the evolution of behaviour in spiders and reveal how molecular evolution drives neuron innovation and the diversification of associated complex behaviours.

## Abstract (web summary)

A brain-cell atlas of a web-building spider and genomic comparisons between web-building and burrowing species identifies preserved ancestral neuron types and candidate neuronal genes that may be involved in the learning and memory pathways underlying web-building and hunting behaviours.

## Main

Spiders are among the most abundant predators with amazing aerial web-building behaviour for prey capture^1,2^. The early ancestors of spiders were probably silk-lined burrow dwellers, and the stereotypical aerial web is believed to be evolved during the Jurassic–Cretaceous period, along with the flourishing of angiosperms and flying insects^3–5^. Such a remarkable behavioural change must have arisen through the evolution of the underlying neural system^6–8^. Yet advances in understanding the mechanisms of how neural systems change over evolutionary timescales have lagged behind our knowledge of behavioural evolution in spiders^9,10^.

The crucial first step for understanding the neural system’s evolution is to identify conserved or novel neuron types, which has been technically challenging for non-model species^11,12^. Recently, high-throughput single-cell transcriptomic approaches have been proven to be a powerful tool for dissecting cell diversity^13,14^ and comparing homologous cell types^15^ with minimal prior knowledge. In addition, although changes in behaviour are often the most obvious outcome of neuronal evolution, all changes must first occur at the DNA level^16^. Integrated multi-omics approaches, including comparative genomics and single-cell transcriptomics, thus are needed to bridge the gap between molecular evolution and cellular diversification^10^.

Here we built a comprehensive atlas of cell types for the adult spider brain using *Hylyphantes graminicola* as a model system and sequenced two genomes (*Atypus karschi* and *Luthela Beijing*) from plesiomorphic burrowing spiders for genome comparisons. We first identified spider-specific neurons and common cell types between spiders and *Drosophila*. Second, we identified ancient gene retention and duplication events in *H. graminicola* and linked cellular novelty with genetic novelty. Third, by utilizing 14 genomes covering the major lineages of Arachnida, we tested how gene family evolution and gene selection jointly shape neuron specificity and contribute to aerial web-building behaviour. Fourth, we used RNAi experiments to test the effect of candidate genes on web building. Together, multi-omics approaches combined with an RNAi-mediated behaviour assay in this study will open a new door to examine the evolution of the unique web-building behaviour of spiders.

## Results

### Transcriptional types of spider brain cell

**Fig. 1:**
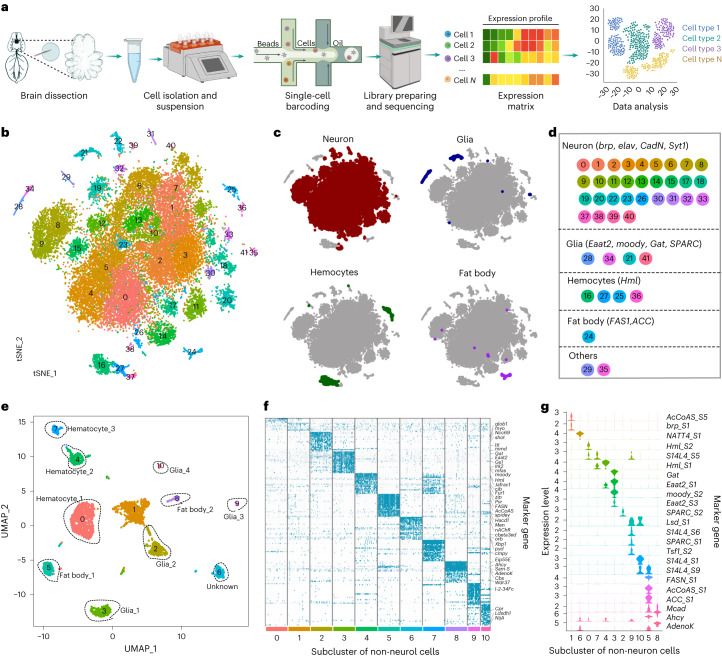
A single-cell atlas of the brain from *Hylyphantes graminicola*. **a**, Scheme of the single-cell study design. **b**, t-distributed stochastic neighbour embedding (t-SNE) plot of the 42 cell clusters generated by grouping the 30,877 cells obtained from the brains of five biological replicates, colour-coded for different cell clusters. Each dot represents one cell. **c**, Four major cell types in the spider brain. **d**, Markers used for annotating the four major cell types and cell clusters in each cell type. **e**, Uniform manifold approximation and projection (UMAP) plot of non-neuron cells. Eleven clusters (0–10) were obtained at a resolution 0.4. **f**, Gene expression heat map of the top 20 marker genes for 11 non-neuron clusters. Colour scale: blue-green, high expression; light grey, low expression. **g**, Violin plots of the expression of selected markers for 11 non-neuron clusters.

**Extended Data Fig. 1:**
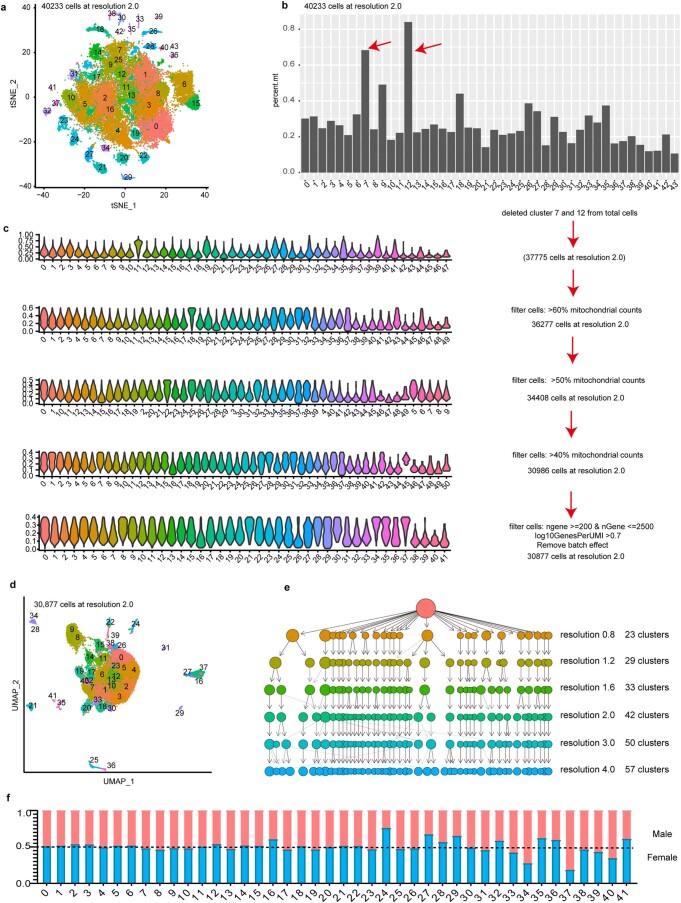
Quality control of the single-cell atlas from spider brain. **a**, t-SNE plot of the 44 cell clusters generated by grouping the 40,233 cells obtained from brains of five biological replicates. Color-coded for different cell clusters. Each dot represents one cell. **b**, The percentage of reads that map to the mitochondrial genome (percent.mt) in each cell cluster. Clusters 7 and 12 with >50% mitochondrial percentage were removed in subsequent analysis. **c**, Clustering and mitochondrial percentage under different parameter settings.at resolution 2.0. **d**, UMAP plot of the 42 cell clusters generated by grouping the 30,877 cells in the final dataset. **e**, Comparison of different cluster resolutions. **f**, The relative abundance of each cell type in different sex. Source data

**Extended Data Fig. 2:**
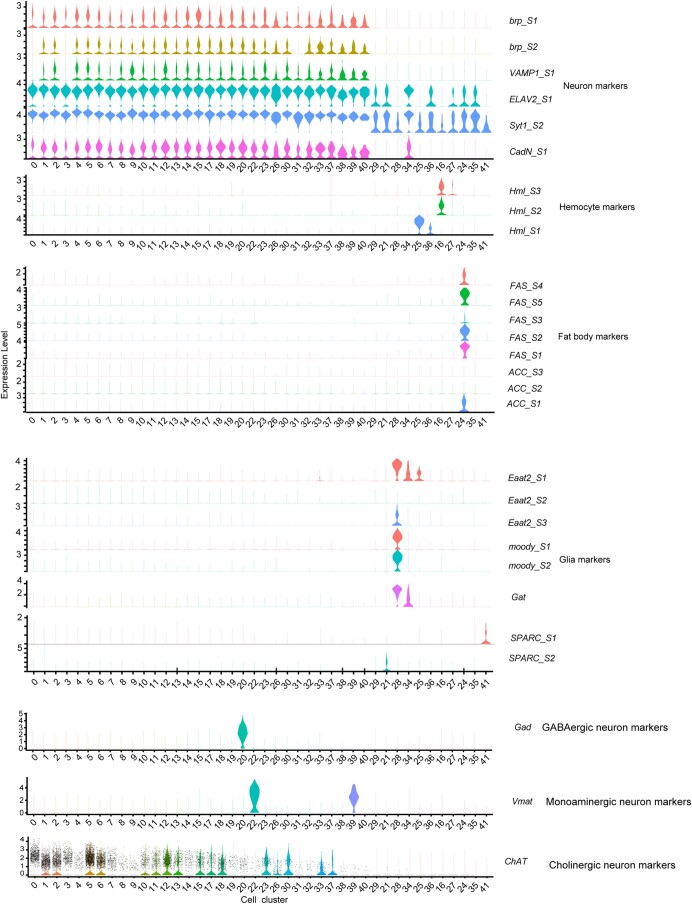
Violin plots of the expression of markers for different cell clusters. Based on these markers, we could identify the cell clusters of neurons (clusters 0-15, 17-20, 22, 23, 26, 30-33,37-40), hemocytes (clusters 16, 27, 25, 36), fat body (clusters 24), glial cell (clusters 21, 28, 34, 41), GABAergic neurons (cluster 20), monoaminergic neurons (clusters 22 and 39), and cholinergic neurons, respectively.

We performed single-cell RNA sequencing (RNA-seq) of spider brains using 10x Genomics technology from adult females (three replicates) and males (two replicates) (Fig. 1a and Supplementary Table 1). A total of 30,877 cells were retained and 42 cell clusters were obtained (Fig. 1b) after quality control (Extended Data Fig. 1 and Supplementary Table 2). Among them, 31 clusters were annotated as neurons by examining the expression of four neuronal markers (*brp*, *elav*, *CadN*, *Syt1*) (Fig. 1c,d and Extended Data Fig. 2). Using multiple non-neuronal markers (Supplementary Table 3), we could identify the cell clusters of hemocytes, fat bodies and glial cells (Extended Data Fig. 2). To better illustrate the cell subtype of non-neuronal clusters, we re-clustered the non-neuronal cells (Fig. 1e,f). Three hemocyte clusters were identified using two *Hml* genes (Fig. 1g). Two *Mcad*-positive clusters were recognized as pericerebral adult fat masses (*Ahcy*, *AdennoK*) and adult fat bodies (*ACC*, *FASN*) (Fig. 1g). Glial cells were usually defined by the expression of *repo*, *pnt, Bdl* or *GLaz*^13,17^, but these genes did not have high cell type specificity in the spider. We then used glial subtype markers and identified four glia types (Fig. 1g): glia_1 (*moody*, *Gat*, *Eaat2*), glia_2 (*SPARC_1*), glia_3 (*Tsf1*, *SPARC_2*) and glia_4 (*SCD5, SEC14L5*). In addition, using markers related to neurotransmitters (Extended Data Fig. 2), we identified the GABAergic (γ-aminobutyric-acid-releasing neuron, *Gad1*, cluster 20), monoaminergic (*Vmat*, clusters 22 and 39) and a large amount of cholinergic neurons (*ChAT*).

### Coexistence of octopamine and norepinephrine in spiders

**Fig. 2:**
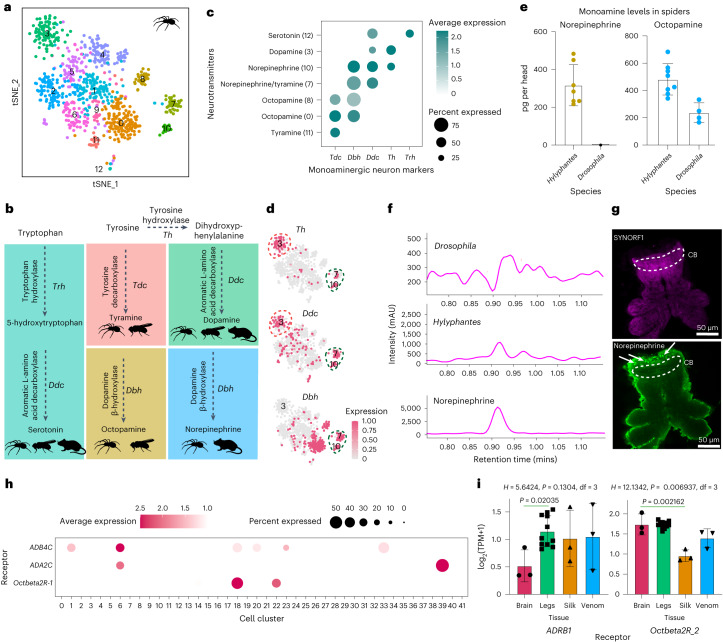
Monoaminergic neurons in spider brains. **a**, Re-clustering of monoaminergic neurons. **b**, Biosynthesis of serotonin from tryptophan and biosynthesis of tyramine, octopamine, dopamine and norepinephrine from tyrosine. **c**, Expression of enzymes that synthesize monoamine neurotransmitters. Six monoaminergic sub-clusters were identified, representing dopaminergic (cluster 3), tyraminergic (cluster 11), octopaminergic (clusters 0 and 8), serotonergic (cluster 12) and noradrenergic neurons (cluster 10). Colours represent the average expression level of a gene and dot sizes represent percentages of cells within each cluster expressing that gene. **d**, Expression pattern of genes that involved in synthesis of dopamine (*Th* and *Ddc*, red circle) and norepinephrine (*Th*, *Ddc* and *Dbh*, green circle). **e**, Norepinephrine (*n* = 7 for *Hylyphantes*, 4 for *Drosophila*) and octopamine level (*n* = 8 for *Hylyphantes*, 4 for *Drosophila*) in *Hylyphantes* and *Drosophila* head, measured by high-performance liquid chromatography (HPLC). Each dot represents the value of a biological replicate. Bars are mean ± standard deviation (SD) across replicates. **f**, Norepinephrine was detected by HPLC. The retention time of *Hylyphantes*, *Drosophila* and standard norepinephrine was present. mAU, milli-absorbance units. **g**, Anti-synapsin (SYNORF1, magenta) and anti-norepinephrine (green) showed the cell bodies (arrow) of noradrenergic neurons were distributed above the central body (CB) of the spider brain. Four biological replicates were performed to confirm the distribution of norepinephrine neurons. Please see the major brain structure of spider in Extended Data Fig. 3. **h**, Expression of representative adrenoceptors and octopamine receptors across different cells in the spider brain. **i**, Expression of representative monoaminergic receptors across different tissues in the spider (*n* = 3 for brain, 11 for legs, 3 for silk glands and 3 for venom glands). One octopamine receptor (*Octbeta2R-2*) was highly expressed in the brain and legs, and one adrenergic receptor (*ADRB1*) was highly expressed in peripheral tissues. Statistical comparisons were performed by Kruskal Wallis test followed by post-hoc Dunn’s correction. *H* is the test statistic for the Kruskal Wallis test and df is freedom degrees. Data in bar plots are mean ± SD. Source data

To characterize cell subtypes of monoaminergic neurons, clusters 22 and 39 were re-clustered and resulted in 13 distinct sub-clusters (Fig. 2a). Using the genes that encode the enzymes for the synthesis of different neurotransmitters (Fig. 2b), we identified the major monoaminergic subtype: tryptophan hydroxylase (*Trh*) for serotonin-producing neurons (Fig. 2c), tyrosine decarboxylase (*Tdc*) and tyramine β hydroxylase (*Tbh*) for octopaminergic neurons and tyrosine 3-monooxygenase (*Th*) and *Ddc* for dopaminergic neurons. One cluster which only expressed *Tdc* was considered as tyraminergic neurons. Surprisingly, we found that cells from sub-clusters 7 and 10 maintained complete norepinephrine synthesis pathway, which suggested that spiders may also have invertebrate-specific norepinephrine neurons (Fig. 2c,d).

**Extended Data Fig. 3:**
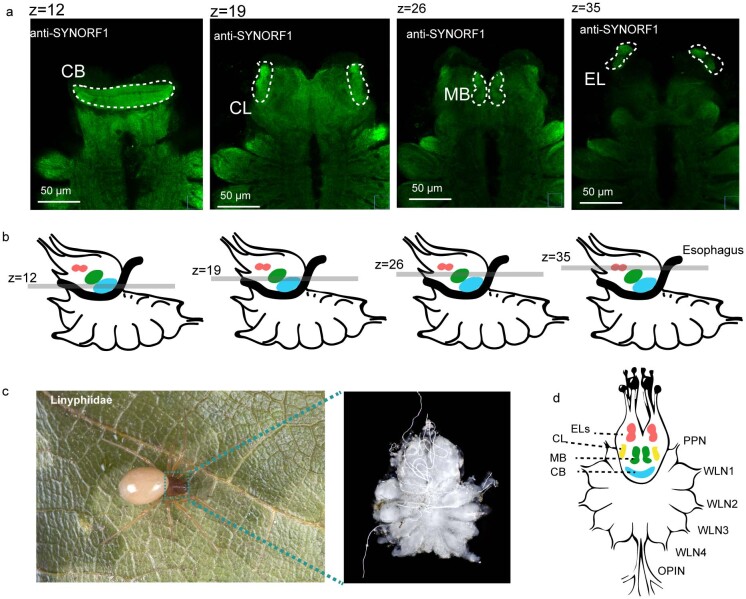
An overview of the central nervous system (CNS) of the spider. **a**, different z-section layer showed the different brain part by Synapsin (anti-SYNORF1, green) staining. **b**, Schematic diagrams showing the organization of spider brain in side view. grey line with different z value corresponding the z-section of confocal laser scanning. **c**, Photograph of an adult male *Hylyphantes graminicola* and the brains used for immunolabeling. **d**, Schematic diagrams showing the organization and major structure of the spider CNS in dorsal view. EL, eye laminae (red): CB, central body (blue); CL, corpus lamellosum (yellow); MB, mushroom body (green); PPN, pedipalpal neuropil; WLN1-4, walking leg neuromere 1-4; OPIN, opistosomal nerves. During dissection, staining, and slide preparation, protocerebrum (including EL, CB, CL, MB) and subesophageal mass (including WLN) are fragile and only with loose connection. So they are almost at same horizontal level and the WLN thus are visible at different z-section.

**Extended Data Fig. 4:**
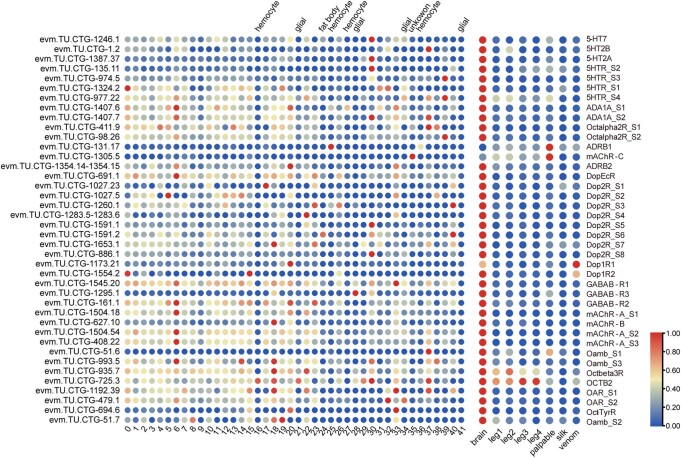
Expression patterns of neurotransmitter receptors across the 42 brain cell clusters and five different tissues. Expression level was scaled to 0-1. Most receptors are highly expressed in brain/neurons. ADRB1 was highly expressed in non-brain tissues and hemocytes (cluster 25). One GABAB receptor (GABAB-R3) was highly expressed in glial cluster (cluster 28). Source data

Octopamine and norepinephrine are chemically and function similarly in invertebrates and vertebrates, respectively^8^. The results of high-performance liquid chromatography (HPLC) revealed the concentrations of norepinephrine and octopamine in the brain of *H graminicola* were 316.7 ± 49.99 pg per head and 481.2 ± 40.48 pg per head respectively (Fig. 2e,f). Norepinephrine immunostaining showed that these neurons were distributed above the central body (CB) of the spider brain (Fig. 2g, brain structure in Extended Data Fig. 3). In addition, adrenergic receptors and octopamine receptors were expressed in different clusters (Fig. 2h) and different tissues (Fig. 2i and Extended Data Fig. 4). The distinctive characteristic of noradrenergic and octopaminergic systems in spiders suggested that octopamine signalling in invertebrates and adrenergic signalling in vertebrates are not equivalent or homologous at least from an evolutionary point of view.

### Expanded peptidergic neuron types in the spider brain

**Fig. 3:**
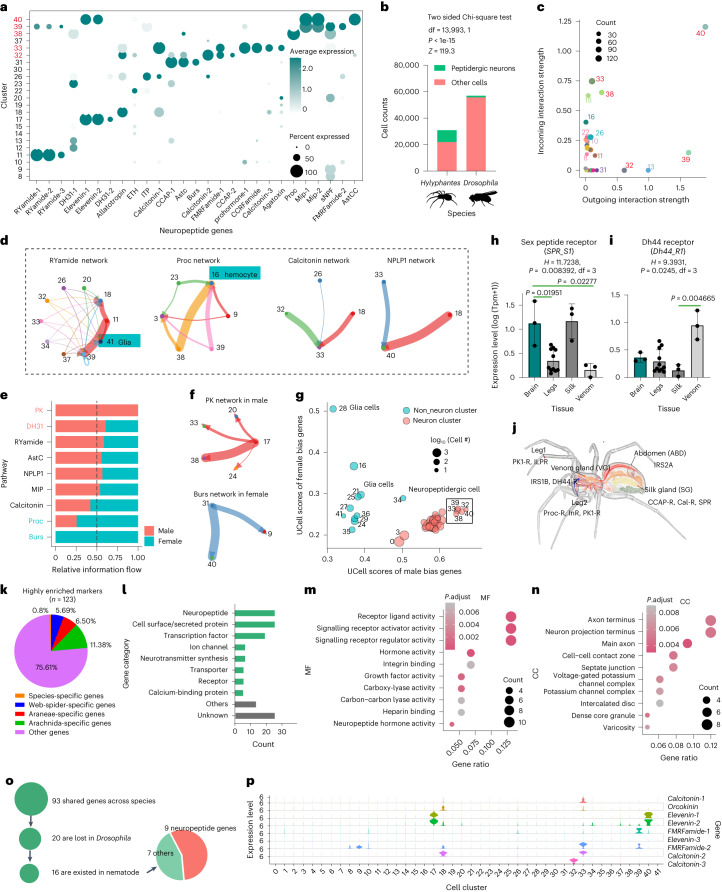
Expanding peptidergic neurons and retention of ancestral neuropeptide genes in spiders. **a**, Dot plots showing the expression of neuropeptides distinguished 23 neuron clusters. **b**, Comparison of proportion of peptidergic neurons between *Hylyphantes* and *Drosophila*^13^. Significance was calculated using two-sided Chi-square test. **c**, The outgoing and incoming interaction strength for each cell inferred by CellChat. The cluster IDs marked by red in **a** and **c** represent five neuropeptidergic neurons. **d**, Four representative neuropeptide networks in spider brains. Lines of the same colour indicated signals sent from the same source cells in each network. Line thickness represents communication strength. Non-neuronal cells communicated with neurons using distinct pathways. For example, glial cell (cluster 41) and hemocyte cell (cluster 16), respectively, received signals from clusters 11 (*RYa*) and 38 (*proctolin*). **e**, Comparison of the overall information flow of each signalling pathway between males and females. Significantly different signalling pathways were coloured red (male) and blue (female). **f**, Male bias neuropeptide network (PK) and female bias neuropeptide network (Burs) in spider brains. **g**, Ucell scores of male bias genes and female bias genes. **h**,**i**, Neuropeptide receptors that are significantly highly expressed in other tissues. Statistical comparisons were performed by Kruskal Wallis test followed by post-hoc Dunn’s correction. Data in bar plots are mean ± SD (*n* = 3 for brain, 11 for legs, 3 for silk glands and 3 for venom glands). **j**, Illustration of the neuropeptide receptors that are expressed in different tissues. **k**, The proportion (group-specific or shared genes) of highly enriched genes in neuronal clusters. **l**, Gene category of the highly enriched genes in neuronal clusters. **m**,**n**, Gene ontology enrichment analysis of the highly enriched genes. MF, molecular function (**m**); CC, cellular component (**n**); only the top 10 were shown, ranked by FDR. **o**, Sixteen of 93 genes are lost in *Drosophila* but exist in both spiders and nematodes, and nine of these genes are neuropeptide genes. **p**, Violin plots showing the expression of nine neuropeptide genes. Source data

**Extended Data Fig. 5:**
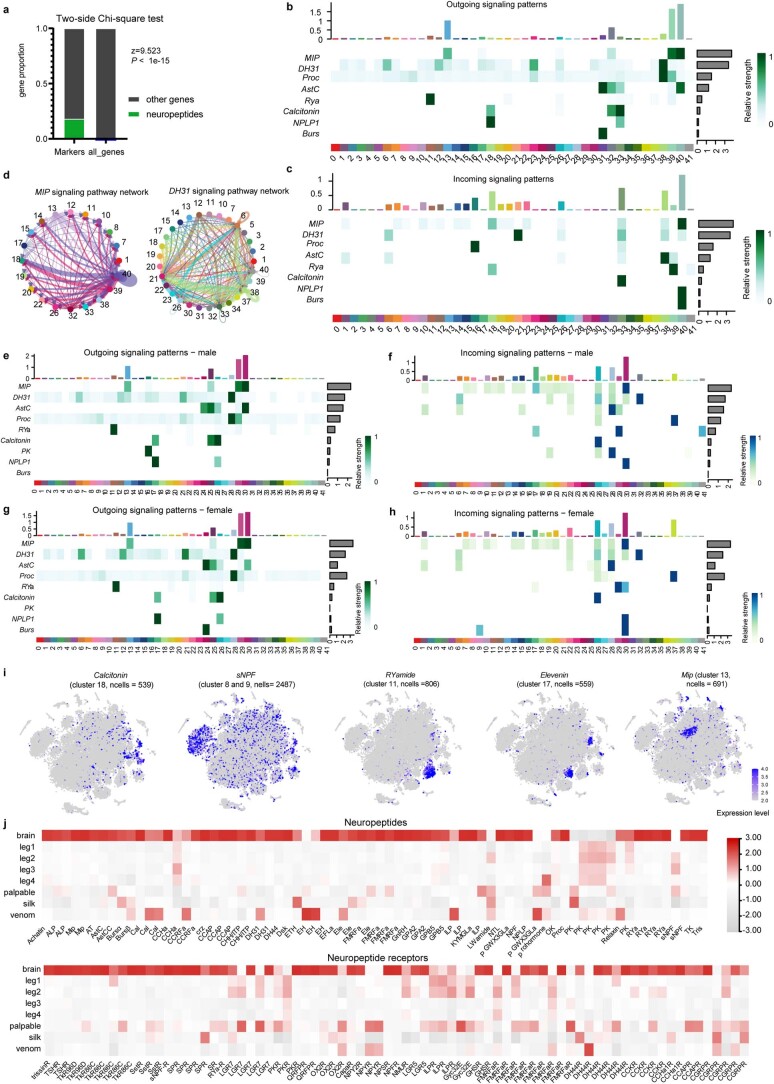
Neuropeptide signaling in spiders. **a**, Comparison of proportion of the gene number of neuropeptides between marker gene list and all genes. Significance was calculated using two-sided Chi-square test. **b-c**, The major outgoing (b) and incoming (c) neuropeptide signals across the 42 brain cell clusters. **d**, The communication networks of two dominant neuropeptide signals (*Mip* and *DH31*). **e-h**, Cell communications strength in male and female of spiders. Cell communication patterns between males and females are similar. But several signals showed sexual difference. **i**, Examples of large clusters (ncells >500) that expressed neuropeptides**. j**, Expression patterns of neuropeptides and neuropeptide receptors across different tissues. Expression level was column-scaled for comparisons. Source data

Genes of neuropeptides were significantly (Chi-square test, *p* < 0.0001) over-represented as cluster markers (Extended Data Fig. 5a). Specific neuropeptides or combinations of different neuropeptides could distinguish a large part of neuron types (Fig. 3a). These include five peptidergic neurons (clusters 32, 33 and 38–40) expressing more than three neuropeptides and multiple unique peptidergic neurons. The proportion of neuropeptide-positive cells (8,979 cells, the average numbers of unique molecular identifier (nUMI) of neuropeptides >100) seems much larger than that in the single-cell atlas of the *Drosophila* brain (Fig. 3b), which only has ~1,000 cells expressing neuropeptides (nUMI > 100) among 57k cells^13^.

We next explored the role of expanding peptidergic neurons in the spider neuron organization by assessing the putative cellular communication mediated by homologue neuropeptide^18,19^ using CellChat^20^ (Extended Data Fig. 5b–d). The dominant neuropeptide sender and receivers were cluster 40 (Fig. 3c), where *Mip*-*SPR* signals contributed most to outgoing and incoming signalling (Extended Data Fig. 5d). Peptidergic neurons showed either higher outgoing (clusters 32, 39) information or higher incoming information (cluster 33). Different non-neuronal cells communicated with neurons using distinct pathways (Fig. 3d). Relative abundance had no significant difference in most neuron types between female and male brain samples (Extended Data Fig. 1f). However, the proportion of peptidergic neurons in male spiders was higher than that in female spiders. The expression level and communication strength of many neuropeptides showed a strong sex-biased pattern (Fig. 3e–g and Extended Data Fig. 5e–h). In addition, we found that the genes that were highly expressed in males were largely expressed in neuropeptidergic neurons (Fig. 3g), suggesting that neuropeptide signals may be highly correlated with sexual behaviour.

Several highly expressed neuropeptides were not captured by CellChat, which may regulate neuronal activity in other tissues. We then used the *τ* index^21^ to define gene specificity by comparing 24 transcriptomes from multiple tissues. Most neuropeptides are strongly expressed in the brain (Extended Data Fig. 5j), but several neuropeptides (for example, *Calcitonins*) and many receptors have a low brain specificity (<0.8) or were strongly expressed in other tissue (Fig. 3h–j and Extended Data Fig. 5j). For example, silk glands also highly expressed *SPR* (Fig. 3h). Several studies showed that mated female spiders produce egg sacs by tubuliform glands, with stronger aggressiveness than virgins^22^. The higher expression level of *SPR* in both brain and silk glands may suggest that *MIP*-*SPR* also plays an important role of postmating response in spiders similar to insects^23^. Together, these results suggested a high informative role of neuropeptides in encoding cell identity and a global role of neuropeptide signals on cell communications in the central nervous system (CNS) and neuron signal conduction from the brain to other tissues of spiders.

### Genetic drivers for cell diversity in spider

To understand the drivers of neuron diversity in the spider CNS, we collected highly enriched marker genes in each neuron type, reflecting both high expression (maximum avg_log2FC ≥ 1) and high specificity (only as markers in ≤five clusters). Most highly enriched marker genes were homologues shared by invertebrates (Fig. 3k). The top three categories of marker genes were neuropeptides, transcription factors (TFs) and cell surface protein/secreted protein, accounting for 40% of the total (Fig. 3l). Gene ontology (GO) analysis showed genes are mostly enriched in receptor activity (Fig. 3m) and axon terminus (Fig. 3n), as expected. Among the 93 invertebrate-shared genes, 16 were lost in *Drosophila* but were still preserved in spiders and nematodes (Fig. 3o). Interestingly, nine of these genes were neuropeptide genes. For example, *DH31* from *Drosophila* is considered a homologue of the vertebrate neuropeptide *calcitonin* gene-related peptide^24^. Spiders retained not only *DH31* homologous to *Drosophila* (lost in nematodes) but also *calcitonin* homologous to vertebrates (lost in *Drosophila*) (Fig. 3p). This result suggested that the retention of ancestral genes, especially neuropeptides, possibly contributes to expanding peptidergic neurons in spiders.

**Fig. 4:**
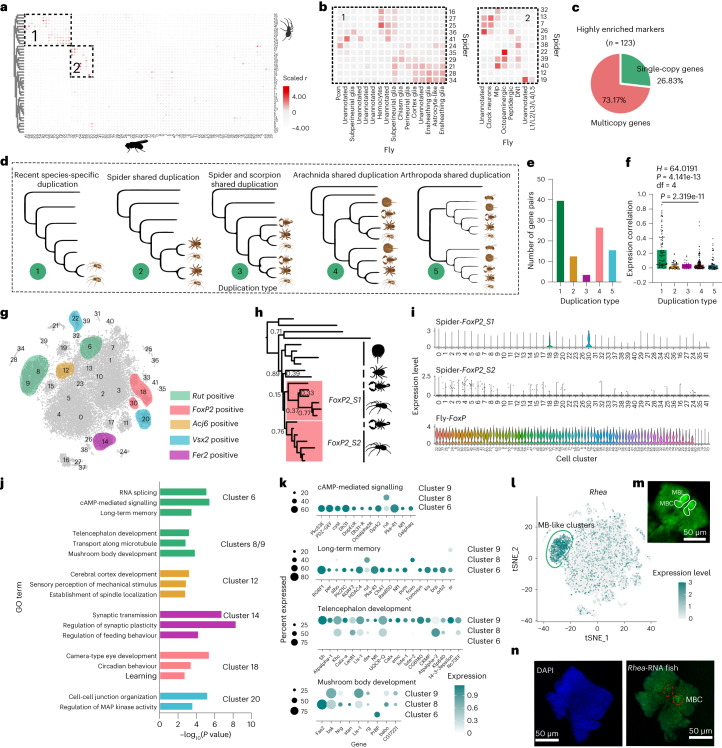
Function annotations of major spider clusters. **a**, Pairwise transcriptional similarity (measured by expression correlation) of cell clusters from *Drosophila* and *Hylyphantes*. **b**, Common cell cluster type between *Drosophila* and *Hylyphantes*. **c**, Proportion of multi-copy gene families in neuronal marker genes. **d**, Gene trees that represent different gene duplication events. **e**, Number of different duplication events in orthogroups of highly enriched marker genes. **f**, Pearson correlations of expression level (log (read count)) of duplicated genes (*n* = 67, 28, 12, 128 and 74 gene pairs for duplication type 1, 2, 3, 4 and 5) based on the single-cell expression matrix. Statistical comparisons were performed by Kruskal Wallis test followed by post-hoc Dunn’s correction. Data in bar plots are mean ± standard errors (SEM). **g**, Selected known cell markers and their expression in the spider brain. *rut*: Kenyon cell marker; *Foxp2*: a transcription factor impairs vocal development in humans and songbirds; *Acj6*: optic lobe (T2/T3/T4/T5) marker; *Vsx2*: optic lobe (Pm3/Pm4, TmY8) marker; *Fer2*: Lawf1/2 marker. **h**, Maximum likelihood (ML) tree of *Foxp2* genes from six spiders, two insects and other arthropods. Nodes with <90% bootstrap support are shown in the tree. Two copies of *foxp2* in spiders are probably from a duplication event in the common ancestor of spiders and scorpions. **i**, Expressed divergence of two *Foxp2* genes in spider and *Foxp* expressed patterns in *Drosophila*. **j**, GO enrichment analysis of neuron markers from different clusters. Reported *P* value*s* were from one-sided of Fisher’s exact test with FDR-adjusted using the Benjamini–Hochberg method. **k**, Dot plot showing the expression patterns of mushroom body-related genes in clusters 6, 8 and 9 of the spider brains. **l**, The expression pattern of gene *rhea*. This gene is a marker gene of clusters 8 and 9. **m**, Synapsin (anti-SYNORF1, green) staining showed the structure of mushroom body (MB) in the spider brains. MBL, mushroom body lobes. MBC, mushroom bodycalyx. **n**, 4′,6-diamidino-2-phenylindole (DAPI) staining (blue) and RNA fluorescence in situ hybridization (FISH) for *rhea* (green) mark subset of MBs. Two independent biological replicates were performed to confirm the expression of *rhea* in the brain. Source data

**Extended Data Fig. 6:**
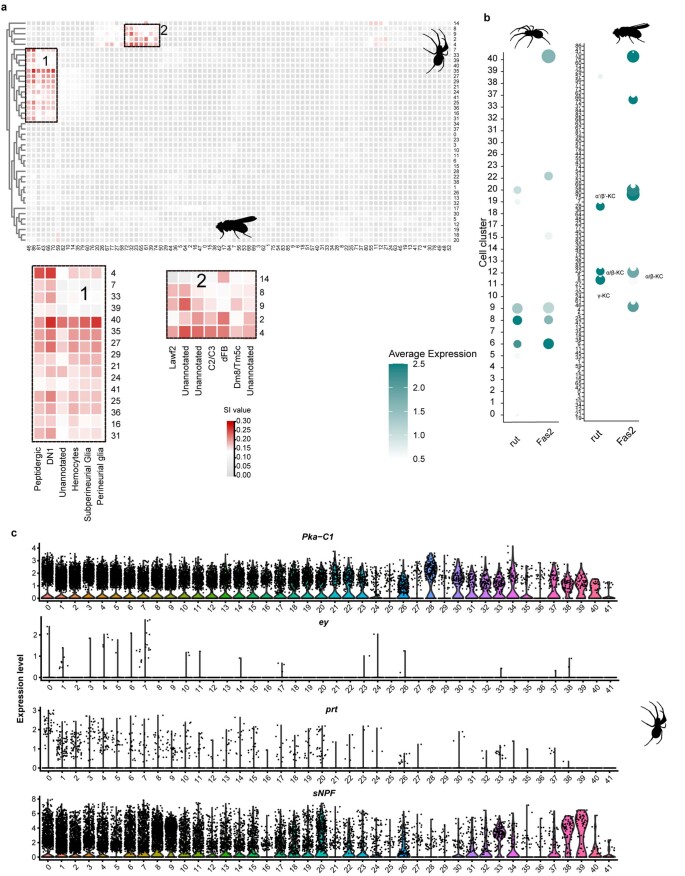
Comparison of transcriptional similarity and expression of selected marker genes between *Drosophila* and *Hylyphantes*. **a**, Pairwise transcriptional similarity (measured by markers similarity index, SI) of cell clusters from *Drosophila* and *Hylyphantes* Red indicates the highest SI value. Dendrogram trees were generated by hierarchical clustering using Ward’s minimum variance method. Numbers 1-2 represented the conserved cell groups between *Drosophila* and *Hylyphantes*. **b**, Dotplot showed the expression pattern of gene rut and Fas2 in *Hylyphantes* and *Drosophila*. **c**, Violin plots of expression of Mushroom body (MB) markers of *Drosophila* in *Hylyphantes*. The MB can be subdivided broadly into three separate groups (α/β, α’/β’, and γ) in Drosophila. Source data

**Extended Data Fig. 7:**
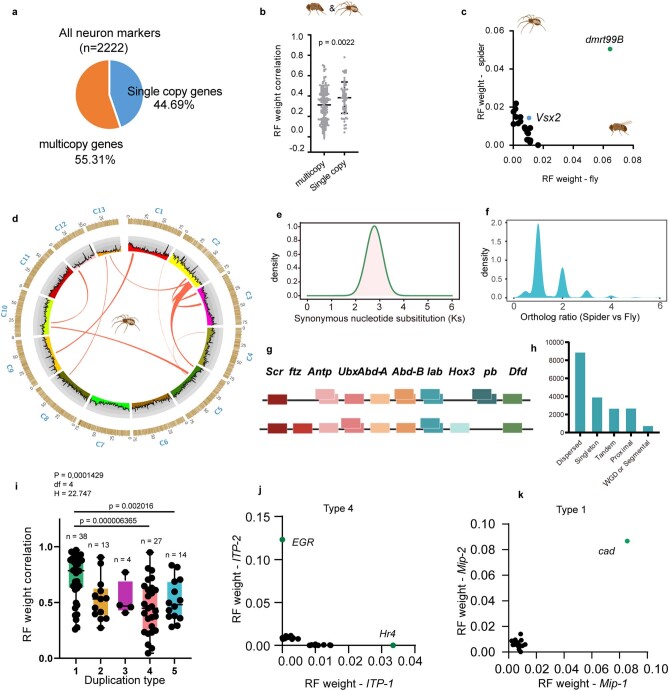
Gene duplication events in *Hylyphantes*. **a**, Proportion of multi-copy gene families among all neuronal marker genes. **b**, Pearson correlations between the Random forest (RF) weights for TF sets associated with each for single-copy (n = 77) and multicopy orthologs pairs (n = 178) between *Drosophila* and *Hylyphantes*. Two-tailed Mann–Whitney U-tests were used to compare difference. Data in bar plots are mean ± SD. **c**, Shared TF (*dmrt99B* and *Vsx2*) of a single-copy ortholog (*Tbh*) between *Drosophila* and *Hylyphantes*. **d**, Genome synteny of *H. graminicola*. **e**, Gaussian fitting curve of the Ks distribution for gene pairs in collinearity blocks. **f**, Distribution of orthology ratios between *Hylyphantes* and *Drosophila* from Orthologous Matrix analysis. **g**, Two copies of HOX clusters in *H. graminicola*. **h**, Gene duplication types of *H. graminicola* that were classified using MCScanX. **i**, Pearson correlations between the RF weights for TF sets associated with duplicated gene pairs of highly enriched genes. Statistical comparisons were performed by Kruskal Wallis Test followed by post-hoc Dunn’s correction. Box plots show minimum to maximum (whiskers), 25–75% (box), median (band inside) with all data points. **j-k**, RF weight for duplicated genes from ancient duplications *ITP* (**h**) and recent species-specific duplication *Mip*. For instance, two *ITP* genes that potentially resulted from ancient duplications showed highly divergent expression patterns and were regulated by different TFs; In contrast, two *Mips* (recent tandem repeats) were expressed in the same peptidergic neurons and regulated by the same TF *cad* (**i**), Only top 10 of TFs for each gene are shown. Source data

Surprisingly, even though most marker genes have homologous genes in *Drosophila* (Fig. 3k), analysis of the patterns of expression similarity between cell clusters from different species only produced several conserved clusters (for example, *Mip* and peptidergic neurons, Fig. 4a,b and Extended Data Fig. 6). We found most of the marker genes that establish and maintain cell type identity (TFs) or determine wiring specificity (for example, cell surface and secreted molecules) belong to the multi-copy gene families predicted by OrthoFinder (Fig. 4c). Multi-copy gene pairs tend to share fewer TF genes and have lower TF weight correlations than single-copy orthologue pairs between species (Extended Data Fig. 7), which suggested that high expression differences of cells between species may result from expression shifts after gene duplications^25^.

We detected two *Hox* clusters and substantial 1:2 paralogy (that is, two copies in spiders and one in flies) in the spider, indicating whole-genome duplication (WGD) in *H. graminicola* (Extended Data Fig. 7). By analysing the phylogenetic topology of the species tree and gene trees (Fig. 4d), we found that recent species-specific duplications and ancient arthropods or arachnids shared duplications were the dominant duplication types for the neuron-specific genes (Fig. 4e). Paralogue pairs from species-specific duplication tend to be expressed in the same cell types and show higher expression correlations (Fig. 4f). In addition, recently duplicated gene pairs also were more associated with similar TFs, compared to ancient duplications (Extended Data Fig. 7i). Gene network analysis showed a complex TF-neuropeptide interaction relationship mediated by novel and duplicated genes in spiders (Supplementary Fig. 9). These results suggested that ancient duplication events possibly play a more important role in cell divergence.

### Function analysis of major cell clusters

Given the large number of neuron clusters in spiders that remain unannotated, we performed GO enrichment analysis for the marker genes from each cluster (Fig. 4g–k). Cluster 18 was enriched in circadian behaviour (GO:0048512) and learning pathways (GO:0007612). In particular, *Foxp2*, which encodes a TF and plays an important role in the development of speech and language in humans and other animals with complex acoustic systems (for example, songbirds)^26^, was distinctively expressed in clusters 18 and 30 (Fig. 4h,i). Unlike the ubiquitous expression pattern in *Drosophila*, the high specificity of this gene in certain neurons of spiders suggests a specialized function, potentially in complex sound production^27,28^. In addition, we identified several neuron clusters that may involve feeding behaviour (cluster 14) and cerebral cortex development (cluster 12) (Fig. 4j).

The mushroom body (MB) is a high integrating centre of the arthropod brain and is mainly comprised of Kenyon cells^13,29^. GO functional enrichment found that clusters 8 and 9 were significantly enriched in MB formation (Fig. 4j,k). Genes such as *bsk*, *CG17221* and *rg* that are involved in MB development showed significantly higher expression levels in clusters 8 and 9 (Fig. 4k). In addition, *sNPF*, which was considered as a marker gene for α/β and γ Kenyon cells^30^, also showed relatively higher expression levels in clusters 8 and 9 (Supplementary Table 4). Notably, cluster 6 is the only cell type enriched in long-term memory (LTM) and mRNA splicing (Fig. 4j). Genes in cluster 6 were enriched for biological processes strongly associated with cAMP-mediated signalling (for example, *Plc21C* and *orb2*). Particularly, rutabaga (*rut*), a membrane-bound Ca^2+^/calmodulin-activated adenylyl cyclase responsible for the synthesis of cAMP, is highly expressed in the MB of *Drosophila*^13^ and also restrictedly expressed in clusters 6, 8 and 9 (Extended Data Fig. 6). Additionally, these clusters highly expressed *Fasciclin 2* (*Fas2*) (Extended Data Fig. 6), which is a marker gene of *Drosophila* Kenyon cells^13^. These results suggested that clusters 6, 8 and 9 may have similar function to insect MBs.

We then performed immunohistochemistry and in situ hybridization to confirm our inference (Fig. 4l–n). In situ hybridization with the anti-*rhea* (marker gene of clusters 8 and 9; Fig. 4l) labelling in probe produced a clear signal in the MB-like regions (Fig. 4n). Immunostaining showed that the *Fas2* was also expressed in the brain MB-like regions (Supplementary Fig. 2). Combining the specific expression of MB marker genes and the GO term analysis, we suggested the clusters 6, 8 and 9 possibly are the MB-like clusters.

### Genetic and cellular specificity of web-building spiders

To compare the genomic differences between aerial web-building spiders and other spiders, we generated de novo genome assembly of the two plesiomorphic burrowing spiders (*Atypus karschi* and *Luthela Beijing*) based on more than 30× PacBio HiFi read. The genome sizes were 876.36 Mb (*A. karschi*) and 4.09 Gb (*L. Beijing*), respectively. Our two genomes possess high continuity and accuracy (Supplementary Table 5), which were similar to other spiders^31^.

**Fig. 5:**
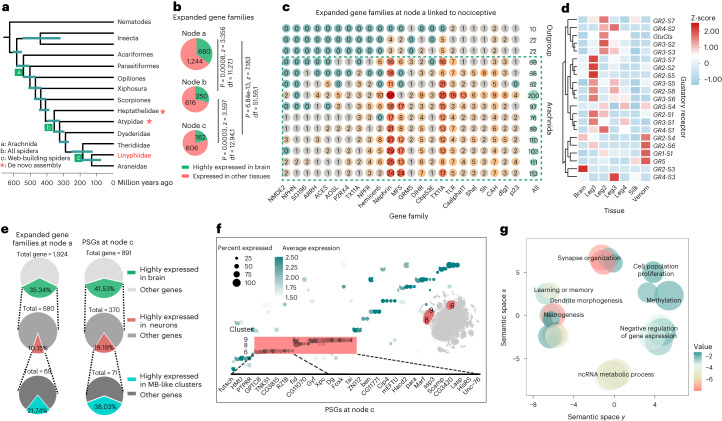
Molecular evolution of web-building spiders. **a**, The maximum likelihood phylogenetic tree containing three web-building spiders, three web-less spiders and other relatives. All nodes received 100% bootstrap support. The de novo genomes that we assembled are marked with asterisks. Linyphiidae were used for the brain single-cell study and are indicated in red. Node a, the common ancestor of Arachnids; node b, the common ancestor of spiders; node c, the common ancestor of Araneoidea. **b**, Expanding gene families in the common ancestor of Arachnids (node a) or the common ancestor of all spiders (node b) were more expressed in the brain than recently expanding gene families (node c). Significance was calculated using two-sided Chi-square test. **c**, Gene families that are involved in nociception. Number in the circles indicates the number of genes in each family. **d**, Row-scaled expression patterns of gustatory receptors in the brain and other tissues. **e**, Comparison of early expanding gene families (node a) and recently positively selected genes (PSGs). PSGs showed higher brain expression bias pattern and preferred expression in mushroom body-like clusters (MB-like). **f**, Dot plot showing expression pattern of PSGs in the common ancestor of web-building spiders. Red shading, genes that were highly expressed in MB-like clusters. **g**, Enriched GO terms of PSGs/REGs. Bubble size represents the number of annotations for a certain GO term in log10 scale, and colour indicates enriched *P* value*s* were from one-sided of Fisher’s exact test with FDR-adjusted using the Benjamini–Hochberg method in negative log10 scale. Significant terms with semantic similarity smaller than default threshold (0.15) are not presented. Source data

By combining two assemblies with 12 published genomes across arthropods, we reconstructed the phylogeny using protein sequences derived from 1,213 single-copy genes (Fig. 5a). Our results consistently show that three aerial web-building spiders form one clade (Araneoidea) and two primitively burrowing spiders are at the basal position of spiders. Ancestral spiders, most likely, were freely roaming hunters^2^, and burrows may represent the ancestral foraging construct^32^. Aerial webs evolved during the late Triassic–Jurassic^1^, coinciding with the explosive diversification of flying insects^33^.

Changes in both web construction and hunting strategy may predict marked changes in the spiders’ genomes, which has facilitated the evolution of spiders at different levels including molecularly, cellularly, in body plans and eventually behaviours. To test this hypothesis, we performed comprehensive genomic comparisons within spiders and their outgroups. We first analysed gene families that changed rapidly in gene number during the evolution process and identified expanded gene families (Viterbi *p* < 0.05) and new emergent gene families in each clade. We found that gene families expanded in the common ancestor of Arachnida (node a) were significantly enriched in neuron-related functions and around one-third of genes were highly expressed in the brain (Fig. 5b). In particular, they were significantly enriched in the feeding and nociceptive pathway (Fig. 5c), which may contribute to the origin of arachnid ancestors’ hunting behaviour. Gene families expanded in the common ancestor of spiders (node b) were also significantly related to brain or neuron function and especially enriched in taste receptor activity (Fig. 5d). Notably, genes expanded at nodes a and b are expressed in various neuron clusters and are not biased towards a particular neuron type (Fig. 5e). In contrast, gene families expanded in the common ancestor of aerial web-building spiders (node c) showed significantly lower brain expression bias than that expanded in the common ancestor of Arachnida (Fig. 5b). In addition, GO enrichments showed that few of these gene families are enriched in neuron-specific pathways. These results suggested the involvement of other evolutionary drivers in neuron evolution specifically for web-building behaviour.

**Extended Data Fig. 8:**
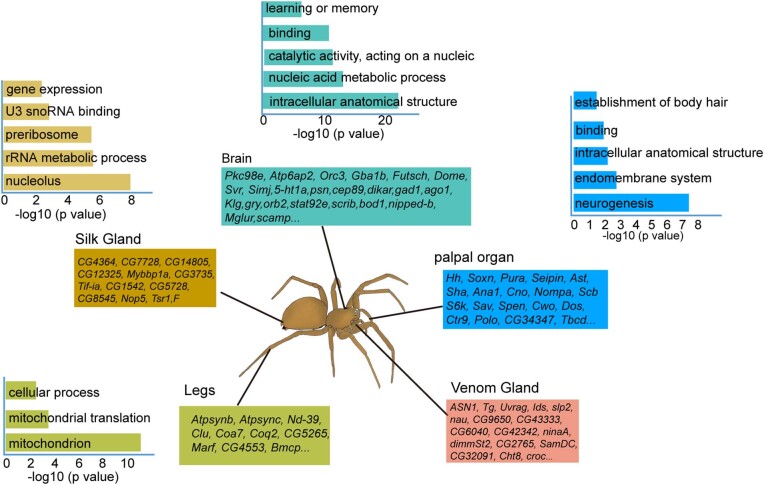
Enriched biological pathways of PSGs and REGs that expressed in different tissues. PSGs: positively selected genes; REGs: rapid evolution genes. p value was adjusted by Benjamini–Hochberg false discovery rate (FDR).

Therefore, we further identified the positive selection genes (PSGs) or rapid evolution genes (REGs) at node c (Supplementary Table 6). Around 42% of PSGs are highly expressed in the brain (Extended Data Fig. 8), and the majority (38%) were enriched (percentage of cells where the gene is detected > 0.25 and average expression > 2) in MB-like clusters (clusters 6 and 8 and 9; Fig. 5f). GO enrichment revealed PSGs and REGs were enriched in the learning or memory (GO:0007611, *P* = 6.29E−08). Among 144 genes in learning or memory pathway (https://biit.cs.ut.ee/gprofiler/gost), 31 were PSGs/REGs and 55 were highly expressed in MBs. For example, two PSGs (*ben* and *Scamp*) were highly expressed in clusters 8 and 9 (Fig. 5f). *ben* interacts genetically in both synaptic transmission and LTM formation with *Scamp*^34^. Mutations of *ben* and *Scamp* could disrupt LTM^34^ and cause a deficiency in odour-associated LTM^35^.

**Extended Data Fig. 9:**
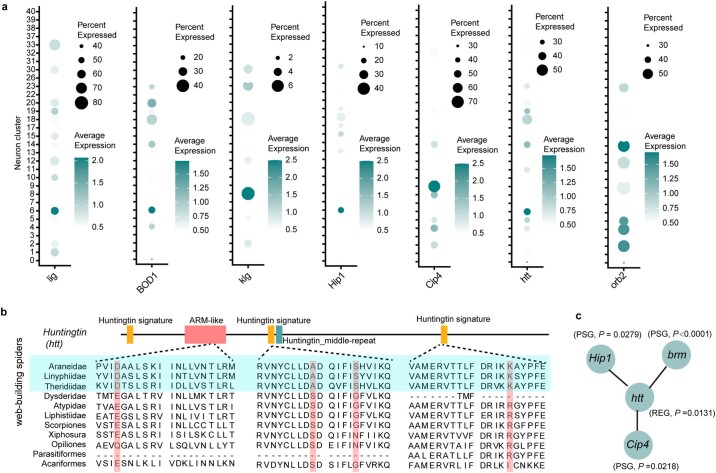
Expression of selected memory-related genes in mushroom body-like clusters. **a**, Dotplot showed the expression of selected memory-related genes in *Hylyphantes*. All genes shown here are positive selection genes (PSGs) or rapid evolution genes (REGs) at the node of the common ancestor of aerial web-building spiders. **b**, Amino acids alignment of huntingtin (*htt*) gene. Protein motifs and domains were annotated by searching InterPro. Red boxes represent amino acids are fixed in web-building spiders. **c**, *htt* and three other PSGs/REGs that have been proven to be interacted with *htt*.

Synaptic change is considered the first step in a series of events that link molecular activity at the synapse and the subsequent intracellular biochemical cascades and cellular changes to the cognitive aspects of memory^36^. We found that PSGs/REGs are significantly related to dendrite development (*P* = 0.0062) and synapse assembly (*P* = 0.0284) (Fig. 5g). Interestingly, *huntingtin* (*htt*), which is required for the formation of the CNS^37^, is a rapidly evolved gene and highly expressed in cluster 6 (Extended Data Fig. 9). This gene is linked to Huntington disease^38^ and involved to long-term synaptic plasticity^39^. In addition, several PSGs/REGs were predicted as *htt*-interacting proteins (for example, *Cip4* and *Hip1*; Extended Data Fig. 9). Ultimately, PSGs/REGs are linked to synapse-specific molecular and biochemical changes, including microtubule binding (*futsch*, *P* = 0.0001), phosphorylation (*BOD1*, *P* = 2.21E-10), regulation of synaptic receptors (*klg*, *P* = 0.0007) and synaptic growth (*orb2*, *P* = 0.0067), which results in changes of synaptic efficacy that may form the neurobiological basis of web-building behaviour.

**Fig. 6:**
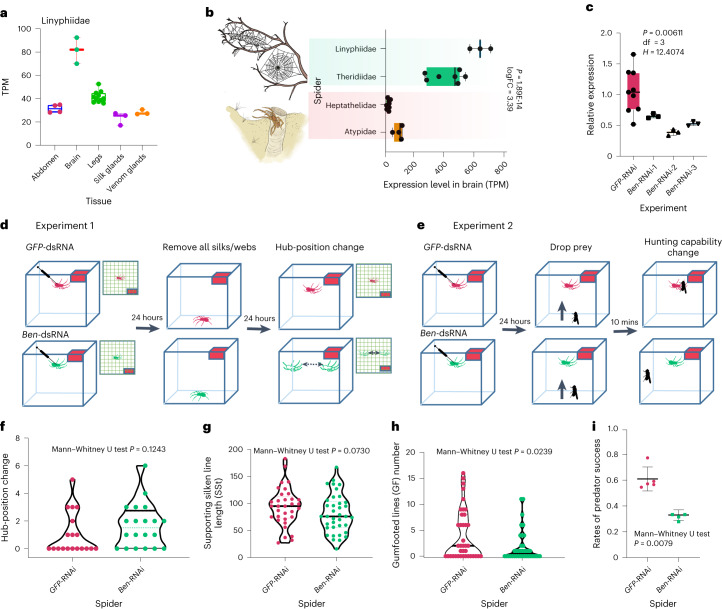
Functional experiment of gene *ben*. **a**, The expression level (transcripts per million, TPM) of gene *ben* across different tissues for *Hylyphantes* (*n* = 4 samples for abdomen, 3 for brain, 11 for legs, 3 for silk glands and 3 for venom glands). **b**, Gene *ben* showing up-regulated expression in web-building spider (*n* = 3 samples for Linyphiidae, *n* = 7 for Theridiidae) brains compared to burrowing spider brains (*n* = 8 for Heptathelidae and 4 for Atypidae). The log fold change (logFC) and significance between different predation strategies were calculated using two-sided Fisher’s test by EdgeR. Reported *P* value is FDR-adjusted using the Benjamini–Hochberg method. **c**, The expression level of *ben* was significantly reduced after dsRNA injection. Statistical comparisons were performed by Kruskal Wallis test followed by post-hoc Dunn’s correction. The green, yellow and blue bars represent three RNAi experiments. **d**,**e**, RNAi experiment to test the effect of *ben* knockdown on the web-building and prey capture capability. **d**, We removed all silk/webs in the plastic box after 24 h upon dsRNA injection and observed whether they would build webs in the same position. **e**, We dropped a fruit fly on the spider’s web after 24 h upon dsRNA injection and allowed the spider to perform its normal prey capture behaviour. After 10 minutes, we removed all the live fruit flies. **f**, Relative hub-position change of spider re-building web. *ben*-RNAi spiders (*n* = 19) showed slightly higher positional change than the control (*n* = 20). **g**, Comparison of supporting silken line length (SSt) between *ben-*RNAi (*n* = 33) and *GFP* control (*n* = 37). **h**, Comparison of gumfooted lines (GF) number between *ben-*RNAi (*n* = 39) and *GFP* control (n = 38). **i**, *ben*-RNAi (*n* = 5) spiders showed significantly lower capture success rate than the control (*n* = 5) during five days after RNAi. Box plots (**a**, **b** and **c**) show minimum to maximum (whiskers), 25–75% (box), median (band inside) with all data points. The solid line and dashed lines within each violin plot (**f**, **g** and **h**) indicate median and quartiles, respectively. Bars showed in **i** are mean ± SD across replicates. Statistical significance in **f**, **g**, **h** and **i** are reported using a two-tailed approach. *Ben*-RNAi spiders and control spiders showed in **d**–**i** are coloured in green and pink, respectively. Source data

To test the effects of expression of the PSGs/REGs on spider web-building and predatory behaviours, we knocked down the expression level of *ben* using RNAi (Fig. 6a–i). Gene *ben* is both a PSG and a REG, and it is highly expressed in mushroom body-like neurons. By comparing the expression level between web-building and burrowing spiders using RNA-seq, we found *ben* showed significantly higher expression level in the brain of web-building spiders (Fig. 6b).

More than half of the *GFP*-RNAi (11/19) spiders build their web in the same position 24 hours after we removed all silks, and 6/20 of *ben*-RNAi spiders stayed in their original position. Changes in hub position of *ben*-RNAi were slightly higher than the control (Fig. 6f; *P* = 0.1243). In addition, *ben*-RNAi spiders showed significantly lower prey capture success rate (Fig. 6i; *P* = 0.0079). We then compared the supporting silken line (SSt) length and gumfooted lines (GF) number between GFP-controlled spider and *ben*-RNAi spiders. We found that *ben*-RNAi spiders showed lower GF numbers (*p* < 0.001) in two days after RNAi (Fig. 6h). GFs delay the escape of insects and give a spider more time to subdue its prey. Lower number of GF may lead to a lower prey capture rate.

Web-building spiders have expanded their niche to previously unoccupied aerial spaces, and eventually, spiders displayed a large diversity of learning processes, from habituation to contextual learning, including a sense of sound, space and even numerosity^40,41^. The observed positive selection and rapid evolution of genes in the MBs may build the neuronal basis for adaption to habitat shifts and the evolution of behavioural changes in web-building spiders.

## Discussion

### Norepinephrine and octopamine in the spider brain

A striking feature of the nervous system of *H. graminicola* is the coexistence of norepinephrine and octopamine in spiders (Fig. 2). Early studies also observed a high level of norepinephrine in the CNS of hunting spiders^42^. Spiders may thus represent one of the ancient organisms where all three transmitters coexist (octopamine, tyramine and norepinephrine). Octopamine is involved in various behaviours such as flying, egg laying and jumping in insects^43^. The previous study proved that octopamine causes a persistent increase in the excitability of spider mechanosensory neurons^44,45^, promoting sensitivity to higher frequencies^46^. Our transcriptome data revealed that octopamine receptors were significantly highly expressed in neuron 18 (*foxp2* positive neuron; Fig. 2g) and in the legs (Extended Data Fig. 4). Octopamine thus probably affects spiders’ sensory perceptions and plays a potential role in auditory or sound-based communication systems.

In vertebrates, norepinephrine in the sympathetic system targets many organs and tissues and causes rapid body reactions, for example, triggering the fight-or-flight response^47^. For spiders, the fight-or-flight is one of the most common decision-making processes spanning their life history^40^. Such response requires the integrated participation of many organs and/or tissues. Interestingly, we found that one of the adrenergic receptors was the only gene that was highly expressed across many organs/tissues (Fig. 2h). Norepinephrine in spiders thus probably preserved a similar function as that found in vertebrates. Overall, the difference between octopaminergic signalling and adrenergic signalling in spiders suggested that octopamine signalling in invertebrates and adrenergic signalling in vertebrates is not functionally equivalent or homologous. More comparative studies in molecular evolution and neural circuits are required to improve our understanding of the evolution and function of these neurotransmitter systems.

### Mushroom body evolution in web-building spiders

One of the most important behavioural innovations of spiders is the emergence of aerial web-building behaviour in spiders^3,48^. The unique structural characteristics related to web building, such as silk and mechanosensory sensilla, have garnered increased interest and detailed descriptions. However, only a small number of molecules (for example, neuropeptide) or neurons had been thoroughly described in spiders^49^. We found that most PSGs were highly expressed on the brain, indicating the key role of CNS in the evolution of spiders beyond the structure innovations. The common ancestors of spiders were probably silk-lined burrow dwellers^2^. Subsequent changes in constructing behaviour (from burrows to web building) and spatial niches (from ground to aerial space) expose spiders to more challenging environmental and biological risks^3^. Spiders evolved many special skills to cope with such challenges, for example, web spiders have been shown to memorize the characteristics of a single captured prey, such as the prey type, size and location, and to change web properties as a function of previous prey catches^50,51^. These experience-dependent modifications of behaviour usually require learning and memory processes that are regulated by the MBs^52,53^, which is a higher-order, multimodal sensory integration centre in the protocerebrum of chelicerates and other arthropod lineage^54,55^.

Interestingly, PSGs/REGs were preferentially expressed in spiders’ MB-like clusters. Many of them have been proven to be involved in cognition and nervous system development. RNAi experiments proved one of these genes (that is, *ben*) significantly reduced prey capture success rate and caused abnormal web-building behaviour. Additionally, strong correlations have been established between learning/memory and synaptic plasticity^56^. Many PSGs/ REGs that are involved in the development of the nervous system are highly actively expressed in MB-like clusters. These genes may contribute to synaptic plasticity and memory formation in spiders. Together, the evolution of MBs and memory-related genes may build the neuronal basis for the emergence of web-building behaviour in spiders.

### Genetic drivers of neuron and behaviour innovation in spiders

**Extended Data Fig. 10:**
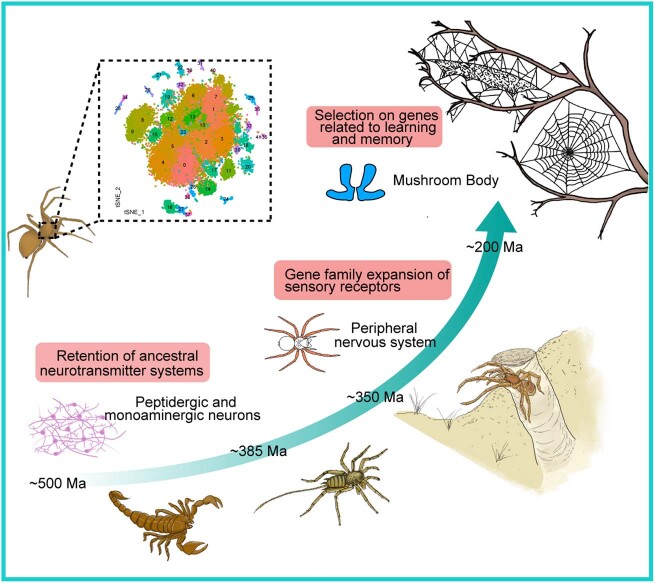
Tracing the genetic drivers of neuron diversity and behavior innovation in the spider. The three key evolutionary steps for web-building emergence are shown: ~500 Ma, the common ancestor of chelicerates; ~350 Ma, at the common ancestors of spiders; and ~200 Ma, at the common ancestors of Araneomorph spiders. Ma, million years ago.

The emergence of web-building behaviour of spiders has gone through at least three key evolutionary steps (Fig. 5a and Extended Data Fig. 10): the appearance of chelicerae in the common ancestor of chelicerates as a raptorial, predatory animal around 500 million years ago (Ma) (ref. ^57^); development of silk-lined burrow constructing behaviour in the common ancestors of spiders around 350 Ma and the evolution of aerial web-building behaviour in the common ancestors of Araneomorph spiders around 200 Ma (ref. ^3^). Our results suggested that a large portion of gene duplications and gene retentions that occurred in the common ancestor of chelicerates potentially contributed to the retention of ancestral neurons (for example, peptidergic neurons) and early neuronal differentiation (Fig. 3). These early genomic changes that are significantly related to feeding behaviour and nociception (Fig. 5c), may have promoted their survival in fierce attack and counter-attack confrontation^58^ in the ancestor of arachnids. The second key step might optimize sensory perception of prey signals through such genetic innovations as the expansion of sensory receptors (Fig. 5d). These changes probably occurred more predominately in the peripheral nervous system than the CNS. The expanding genes that occurred in the common ancestor of web-building spiders were less enriched in the brain (Fig. 5b), and the neuron and behaviour innovation in the third step may have resulted from genes undergoing positive selection or rapid evolution. These genes were preferentially expressed (~38%) in the MBs and related to the function of LTM and synaptic development and may have provided the last critical drive for advanced web-building behaviour.

This study suggested that an integrated multi-omics approach, including genomics, transcriptomics and single-cell transcriptomics, might represent a valuable strategy for evolutionary neuroscience discovery, especially for non-model animals. Our study also has several limitations. First, the cell number in this study may be insufficient to cover all neuron types. The expression characters of the rare cluster were potentially less reliable. The additional dataset with more cell numbers and more experiments could fill some of the gaps. Second, it was still difficult to deeply understand the neuronal circuits underlying the web-building behaviour. Advanced gene editing and neurobiological technology^59^ will greatly improve our understanding of spider web-building behaviour.

## Methods

### Animals for single-cell sequencing

Adult samples of the aerial web-building spider (*Hylyphantes graminicola*) were collected from Anci district, Langfang, Hebei, China (39° 31.90’ N, 116° 38.15’ E) between September and October 2020. Collected spiders used for brain dissection were housed individually in a glass tube (Φ12 mm × 80 mm) at temperature- and humidity-controlled condition (24–26 °C and 50–60% humidity) in a 14 h–10 h light–dark cycle. No ethical approval was needed because spiders used in this study are common species with huge population size in the field and are not threatened species.

### Brain dissection and single-cell dissociation

Five single-cell libraries (two from males and three from females) were constructed for 10× single-cell sequencing. For each library, brains (35–40 mixed-aged adults) were dissected in cold 1× formaldehyde-phosphate-buffered saline (PBS) solution using fine forceps and transferred to a tube containing MACS Tissue Storage Solution. The brain tissue was dissociated into single cells using the Adult Brain Dissociation kit (Miltenyi Biotec number 130-107-677) with these modifications: (1) after termination of the gentleMACS programme, the C-tube with the sample was incubated at 37 °C for 10 minutes; (2) all centrifugations were performed at 220 G for 8 min at 4 °C; (3) myelin debris and erythrocyte removal steps were omitted to prevent loss or bias in the recovered cell yields.

### 10× genomic single-cell sequencing

Single-cell transcriptomic amplification and library preparation were performed at Capitalbio Technology Corporation (Beijing, China). Libraries were made using the Chromium Single Cell 3’ v3 kit from 10X Genomics. Briefly, spider brain single cells were suspended in 0.04% BSA–PBS. Cells were added to each channel to capture the transcriptomes of ~5,000 to 10,000 cells per sample. Cellular suspensions were loaded on a GemCode Single Cell Instrument (10X Genomics) to generate single-cell gel beads in emulsion (GEM). GEMs and scRNA-seq libraries were prepared using the GemCode Single Cell 3ʹ Gel Bead and Library Kit (10X Genomics) and the Chromium i7 Multiplex Kit (10X Genomics), according to the manufacturer’s instructions. Libraries were sequenced on an Illumina Novaseq6000 with a sequencing depth of at least 100,000 reads per cell with pair-end 150 base pair (PE150).

The CellRanger pipelines v4.0.0 provided by 10X Genomics were used to process the sequenced libraries (alignment, barcode assignment and UMI counting). The reference genome was built based on the spider genome released on ScienceDB Digital Repository: 10.11922/sciencedb.01162. The number of cells detected in the experiment was determined by CellRanger based on the number of barcodes associated with cell-containing partitions and estimated from the barcode UMI count distribution. A digital expression matrix was obtained for each experiment with default parameters. From a total of 2,192,024,585 reads, 82.2% were mapped to the *H. graminicola* genome, giving an approximate sequencing depth of 60,000 reads per cell (ranging from 31,483 to 111,407). The median number of genes per cell ranged from 734 to 1,258 (average 1,070). Other sequencing matrices of the spider brain (five samples) of 10X Genomics scRNA-seq are summarized in Supplementary Table 1.

### Raw data processing and quality control

Seurat pipeline v4 (ref. ^60^) was used to perform basic processing and visualization of the scRNA-seq data in R v4.0 (ref. ^61^). The initial dataset contained 40,233 cells from five samples (Supplementary Table 1). Cells with high mitochondria expression levels are considered low-quality cells. For instance, cells with mitochondrial RNA > 20% were removed in studies of the *Drosophila* larval brain^62^. However, the proportion of mitochondrial RNA in our five samples is about 30%. We could not simply specify a parameter set. Therefore, we first used all cells to process and visualize the initial scRNA-seq data using the *vst* method in Seurat. Clusters were identified by the ‘*FindClusters*’ function with a clustering resolution of 2 (see below for details on Seurat cell clustering). We obtained 44 clusters and selectively removed entire clusters with the majority of cells having ≥40% mitochondrial RNA and under 1,000 detected UMIs (Extended Data Fig. 1)^63^. We then set different mitochondrial percentages: 20%, 30%, 40% and 50% to check if the remaining cells still can generate specific clusters with high mitochondrial percentages. Finally, we used the following parameters for the remaining individual cells to exclude outliers: minimum percentage mito = 0, maximum percentage mito = 40%, minimum number of UMI = 500, maximum number of UMIs = 60,000, minimum number of nGene = 250 and maximum number of nGene = 3,000. Additionally, genes expressed in at least three cells were considered for the analysis. More stringent criteria decreased the number of cells included without further improving the clustering. In the final dataset, a median of 1,375 genes and 3,777 unique molecular identifiers (UMIs) per cell were obtained across five replicates (Supplementary Table 2).

### Seurat cell clustering

After initial quality control, a total of 30,877 cells were retained. We then used the R package Seurat for normalization, integration, dimension, reduction, clustering and visualization. Retained cells from each sample were log normalized and scaled with LogNormalize. Variable genes were identified with the *FindVariableFeatures* function (selection.method = ‘*vst*’, nfeatures = 2,000). Sample integration and batch removal were performed using Harmony package v0.10 (ref. ^64^). Clustering and visualization of the data were accomplished using Seurat’s linear dimension reduction (principal components analysis, PCA) followed by t-distributed stochastic neighbour embedding (t-SNE). Clusters with only one cell were removed. Cells were clustered using a resolution from 0.2 to 6 (Supplementary Table 2). A comparison of different cluster resolutions was evaluated with the clustree package v0.5.0 (ref. ^65^). Cluster resolutions above 2 yielded few new clusters and resulted in 42 clusters (Extended Data Fig. 1 and Supplementary Table 2). DecontX^66^ and DoubletFinder^67^ were used to estimate RNA contamination and detect doublet for each cluster (Supplementary Figs. 5 and 6). Differentially expressed genes were found using the *FindAllMarkers* (min.pct = 0.2, only.pos = true) function with Wilcoxon Rank Sum test (Supplementary Table 4).

### Spider gene identification

The cell type can be identified by overlaying the expression of specific marker genes, requiring previous knowledge of gene expression in specific tissues or cells. This prior information is mostly from model species (for example, *Drosophila*). The spider (*H. graminicola*) genes were annotated against the *Drosophila* protein sequences by the reciprocal best hit method using BLASTP in BLAST v2.10.0+ (E-value < 1e−5) (ref. ^68^). This step identified 5,977 homologous genes between flies and spiders.

To account for gene paralogues and gene duplication events, an aggregated table of ‘meta-genes’ was created^69^. Each meta-gene may include all genes homologous to one fly gene. For this purpose, we clustered gene families using OrthoFinder v.2.3.118 (ref. ^70^) under the default parameters (Inference of orthogroups and pairwise orthology relationship sections). This analysis identified 8,026 orthologues between the spider and the fly.

Gene functions were assigned according to the best match to the databases of National Center for Biotechnology Information non-redundant nucleotide database (NCBI-NR, http://www.ncbi.nlm.nih.gov) and Swiss-Prot (http://www.uniprot.org/)^71^ by protein–protein Basic Local Alignment Search Tool (BLASTP, E-value ≤ 1e−5) and the Kyoto Encyclopedia of Genes and Genomes^72^ using Diamond v0.8.22 (E-value ≤ 1e−5) (ref. ^73^). Protein domain identification and Gene Ontology (GO) (http://geneontology.org/) analysis were performed through InterProScan v5.32–71.0 (ref. ^74^). Signal peptide prediction was performed using SignalP v6.0b (ref. ^75^). Homologous transcription factors (TFs) were matched against *Drosophila* using BLASTP. Novel TFs in the spider genome were predicted using DeepTfactor^76^.

### Cell type annotation

We used three methods to determine cell type for each cell cluster: marker genes, expression similarity across species and GO enrichment:

We first used known cell type-specific/enriched marker genes of *Drosophila* that have been previously described to determine cell type (details in Supplementary Table 3). This includes neuronal markers (*brp*, *nSyb*, *elav*, *Syt1* and *CadN*), Glia markers (*moody*, *Eaat2*, *Gat* and *Gs2*), fat body markers (*FASN1*, *ACC*).

We then compared the expression similarity of each cluster between the fruit fly and spider using two approaches: (1) correlations between mean expression profiles among clusters^77^ and (2) marker similarity^25^. Both methods can reduce noise stemming from biological or technical variation across individual cells and treats each cluster as a unit of comparison. For the correlation method, differentially expressed genes for fly^13^ and spider were identified using Seurat *FindAllMarkers*. For each fly–spider cluster comparison, these lists were intersected to identify a common set of BRH genes. Average cluster expression profiles were subsetted and transformed for this common gene set. Cluster expression correlations were calculated using Spearman’s correlation coefficient. For the marker similarity method, we also identified the marker gene list for each cluster from different species. These lists were then used to calculate the marker similarity for each cluster between the fly and spider following the methods from Shafer et. al^25^. The correlations matrix and marker similarity matrix were hierarchically clustered using Euclidean distances and the ‘ward.D’ agglomerative method.

GO functional enrichment analysis of marker genes that were specific to each cell was performed using R package clusterProfiler v3.18.1 (ref. ^78^). The *P* value was adjusted by Benjamini–Hochberg false discovery rate (FDR), and terms with an adjusted *P* value of <0.05 were recognized as significantly enriched. REViGO (http://revigo.irb.hr/) was used to cluster the over-represented GO terms and construct the interactions of terms^79^.

### Cell–cell communication mediated by neuropeptides

We created a neuropeptides and neuropeptide receptors list from the gene annotation results. The ligand–receptor pairs of *H. graminicola* were defined based on the known neuropeptide–receptor pairs in *Drosophila*^18,19^. Potential cell–cell interactions mediated by neuropeptides in different cell types were inferred using CellChat v1.1.3 (ref. ^20^), a tool that can quantitatively infer and analyse intercellular communication networks from scRNA-seq data. We first used all five samples and computed ligand–receptor interaction strengths for all pairs of cell types following the official workflow. For comparison of cell–cell communication between males and females, we ran each dataset (females and males) separately and merged two objects. Sexual difference in interaction strength for each signal pathway was estimated by the Wilcoxon test (*P* < 0.05) in CellChat. Cell clusters with significant changes in sending or receiving signals between male and females and the overall information flow of each signalling pathway were identified following the official workflow.

### Identification of putative GRNs using GENIE3

We used GENIE3 v3.16 to identify TFs that were predictive of the expression of terminal effector genes associated with the functions of brain neuron diversity^80,81^. Briefly, the single-cell expression matrix was subjected to GENIE3 algorithm. Genes that were identified as cell markers were listed in the target genes list. The lists of TFs and their corresponding weights for each target gene were used in downstream analysis. Gene co-expression networks for each TF were constructed by the GENIE3 package. Only weight values more than 0.1 and the top 50 targets for each TF were retained and plotted by Cytoscape v3.7.2 (ref. ^82^).

### Whole-genome duplication event analysis of *H. graminicola*

We used pipelines in the wgdi package v0.1.6 (https://pypi.org/project/WGDI) to perform Colinear block analysis^83^. First, the putative paralogous and orthologous gene pairs within the genome were searched using BLASTP (E-value < 1e−5) with a maximum of 25 alignments. The maximal collinearity gap length between genes was set as 50. Then, synonymous substitutions (Ks) values of identified colinear gene pairs were calculated using YN00 v4.9 (ref. ^84^). Third, Gaussian density fitting was performed to estimate the probability density distribution for median Ks. We also used MCScanX in TBtools v0.665 (ref. ^85^) with default parameters to identify inter-chromosomal colinear blocks of *H. graminicola*. MCScanX was also used to classify genes into five categories, namely singletons (that is, genes without any duplicate), dispersed (duplicates occurring more than ten genes apart or on different scaffolds), proximal (duplicates occurring on the same scaffold at most ten genes apart), tandem (consecutive duplicates) and segmental (block of at least five collinear genes separated by less than 25 genes missing on one of the duplicated regions).

We further identified ten highly conserved Hox genes in arthropods, which are considered to play important roles in the common ancestor of panarthropod^86^. HOX protein sequences of four species (*Daphnia magna*, *Drosophila melanogaster*, *Parasteatoda tepidariorum* and *Tribolium castaneum*) were downloaded from NCBI and Swiss-Prot databases. We performed BLASTP (E-value < 1e−10) to search for the candidates. For ensuring the accuracy of identification, we used MAFFT (multiple sequence alignment software v7.455) with the G-INS-i model (iterative refinement method with consistency and WSP scores)^87^ to observe the clustering between the obtained genes and the downloaded HOX genes. The obtained gene clusters were further manually checked in the NCBI-NR database. Duplicated HOX gene clusters supported the signature of WGD.

### Genome sequencing, assembly and annotation for two plesiomorphic burrowing spiders

Two spiders (*Atypus karschi* and *Luthela Beijing*) were selected for genome sequencing and comparative genomic analysis because they represent the basal lineage of spiders with the primitive hunting behaviour (burrowing). Samples of the purseweb spider (*A. karschi*) were collected from the bamboo forest Tongji, Chengdu, Sichuan, China (31.18° N, 103.84° E). Samples of the segmented spider (*L. Beijing*) were collected from Purple Bamboo Park, Haidian district, Beijing, China (39.94° N, 116.32° E). All samples were starved and reared in the lab for more than 72 hours at room temperature. Genomic DNA for short- and long-read sequencing was isolated from the cephalothorax of adult spiders using the Qiagen Blood and Cell Culture DNA Kit (QIAGEN).

The high-fidelity libraries were sequenced on the PacBio Sequel II system in Circular Consensus Sequencing mode at Novogene Technology Co. PacBio reads were first assembled using wtdbg2 v2.5 (ref. ^88^). The contigs of the assembly were polished by Racon v1.4.17 for three rounds (https://github.com/isovic/racon) using long reads and then by NextPolish v1.4.0 (rerun = 2) using short reads^89^. Gene structure annotation was performed by braker-2.1.6 (ref. ^90^), combining Augustus v3.3 (ref. ^91^) and GenomeThreader v1.7.3 (ref. ^92^). For transcriptome-based annotation, RNA-seq data were mapped to the reference genome using STAR v2.7.3a (https://github.com/alexdobin/STAR). We then identified the coding region of transcripts using transdecoder v5.5.0 (https://github.com/TransDecoder/TransDecoder). All of the predicted gene models above were then combined to create a consensus gene set using EVidenceModeler v1.1.1 (ref. ^93^).

### Behavioural classification of six spiders in this research

Coding spider foraging strategies is notoriously complex and controversial. Here we simplify the foraging strategies of spiders in this study based on web architectures and their habitats^3^: (1) aerial web-building spiders—spiders that use suspended webs that are architecturally stereotyped or relatively amorphous in open aerial habitat, including foliage of shrubs, herbs and trees; (2) burrowing spiders—spiders that live in a burrow with silk lines or rudimentary webs, and these ‘webs’ have few to no direct junctions between discrete silk threads; (3) hunting spiders—no prey capture webs.

### Inference of orthogroups and pairwise orthology relationships

We inferred orthogroups using 14 species, including Nematoda, *Caenorhabditis elegans*, two insects (*Apis mellifera* and *Drosophila melanogaster*), the horseshoe crab *Limulus polyphemus*, three non-spider Arachnida (the opiliones *Phalangium opilio*, the tick *Hyalomma asiaticum* and the mite *Tetranychus urticae*), two plesiomorphic burrowing spiders (*Atypus karschi* and *Luthela sinensis*), one plesiomorphic hunting spider (*Dysdera sylvatica*) and three aerial web-building spiders (*Argiope bruennichi*, *Parasteatoda tepidariorum* and *Hylyphantes graminicola*) with OrthoFinder (-M msa -S blast -T iqtree). Briefly, protein sequences of each species were assigned to homologous families using BLASTP and the clustering algorithm MCL^94^. Multiple sequence alignments were finished using MAFFT with default parameters. We then built orthogroup trees and a species tree using IQTREE v1.6.12 (ref. ^95^).

After inferring orthogroups, we predicted pairwise orthology relationships and characterized the relative times of duplication events for each gene family based on the gene tree topology from IQTREE. We defined five scenarios that corresponded to different duplication events^96^: Hypothesis 1, *H. graminicola*-specific duplication events; Hypothesis 2, common duplication in the most recent common ancestor of spiders; Hypothesis 3, common duplication in the most recent common ancestor of spiders and scorpions (MRCA_spider_scorpion); Hypothesis 4, common duplication in the most recent common ancestor of Arachnida (MRCA_Arachnida); Hypothesis 5, common ancient duplication in the most recent common ancestor of spiders and insects (MRCA_spider_insects).

### Gene expression matrix from different tissues

Twenty-four RNA samples from eight tissues, including the venom gland, brain, silk glands, abdominal tissue without silk glands and leg pairs I–IV from our previous study^31^ were used to determine the tissue-specific expression pattern for each gene. Tissue-specific genes for each tissue were identified using the *τ* index^97^. Briefly, the gene expression levels in the detected tissues were quantified as the transcripts per million (TPM) using the following formula: TPM = (CDS read count × mean read length × 10^6^) / (CDS length × total transcript count). We then used the expression matrix to calculate *τ* tissue-specific expression indices as follows:

$${{\tau }}=\frac{{\sum }_{{{i}}=1}^{{{n}}}(1-{\widehat{{{X}}}}_{{{i}}})}{{{n}}-1}{\rm{;}}\,{\widehat{{{X}}}}_{{{i}}}=\frac{{{{X}}}_{{{i}}}}{\mathop{\max }\limits_{1\le {{i}}\le {{n}}}({{{X}}}_{{{i}}})}$$

*X*_*i*_ is the expression of the gene in tissue *i*, *n* is the total number of tissues. The values of *τ* range between 0 and 1. Values close to 1 indicate completely tissue specific, and values close to 0 indicate ubiquitously expressed genes. We classified genes as cellular-specific expressed when *τ* > 0.8.

### Identification of neuron-specific genes in the spider brain

To better understand the forces that drive gene expression diversity in the spider nervous system, we identified the most highly enriched and highly cellular-specific genes in CNS that are related to neuron clustering and neuron type definition using two methods: marker-dependent and cellular specificity indices. We selected neuron-specific genes from the marker gene list of neuron clusters using two thresholds: relatively higher expression (maximum avg_log2FC ≥ 1) and high specificity (only as markers in ≤ 5 clusters). We defined the orthogroups as neuron-specific orthogroups if any gene in this group was listed in the neuron-specific gene set.

### Calculation of expression divergence of duplicated genes

To explore whether duplicated genes contribute to neuronal diversity, we determined the expression divergence of duplicated genes for the neuron-specific orthogroups. First, we tested whether duplicated genes are expressed more frequently in the same cell type than randomly selected gene pairs; second, we tested whether duplicated genes showed a higher correlation of mean expression level across different cells than randomly selected gene pairs; third, we tested whether duplicated genes shared more similar TFs (calculated as the number of shared TFs from the top 25 TFs resulted from GENIE3) than randomly selected gene pairs; fourth, we tested whether duplicated genes showed higher TF weight correlations than randomly selected gene pairs^25^.

### Gene family expansion and gene selection analyses

To explore the molecular and neuron innovation accompanying aerial web-building and hunting behaviour, we first searched the expanding gene family and selective events that are specific to the aerial web-building spider lineage.

To evaluate gene family expansion and contraction, we used CAFÉ v5.0 (ref. ^98^) with default parameters, which applies the results of orthologue groups from the OrthoFinder programme. The time tree was constructed using MCMCTree framework in PAML (Phylogenetic Analysis Using Maximum Likelihood) v4.9j (ref. ^99^). The gene family was regarded as significantly expanded or contracted (Viterbi *P* ≤ 0.05) when the copy number of focused branch lineages was higher or lower than its ancestral branch lineage, respectively. Gene orthogroups with >100 copies were filtered out. We performed positive selection analysis with six spider species including three aerial web-building spiders, two plesiomorphic burrowing spiders and one web-less hunting spider. We obtained 5,680 best-to-best hits as orthologous genes, which were shared by six species and used for positive selection analysis. Orthologous proteins were aligned by MAFFT. Poor alignments were trimmed with trimAL v1.4.rev15 (-gt 0.9 -st 0.001) (ref. ^100^). We used branch-site likelihood ratio tests in CODEML package of the PAML to identify positive selection genes (PSGs) for the ancestral branch of aerial web-building spiders. The branch-site model allows omega to vary both among sites in the protein and across branches on the tree, aiming to detect positive selection affecting a few sites along particular lineages. We performed the test by comparing two models: the null model (using the settings model = 2, NSsites = 2, omega = 1) and the alternative model (model = 2, NSsites = 2). The likelihood ratio test has degrees of freedom = 1. We used the F3 × 4 codon model of Goldman and Yang^101^ to calculate the equilibrium codon frequencies from the average nucleotide frequencies at the three codon positions (CodonFreq = 2). We obtained FDR with the Benjamini and Hochberg method^102^. Genes with FDR < 0.05 and the ratio of non-synonymous to synonymous substitutions (d*N*/d*S*) in the foreground >1 were considered PSGs.

To examine the rapidly evolved genes (REGs), branch model (model = 2, NSsites = 0) was used to detect ω of foreground branch (ω0), average ω of all the other branches (ω1) and the mean of whole branches (ω2). Then a χ2 test was used to check whether ω0 was significantly higher than ω1 and ω2 under the threshold FDR < 0.05, which hinted that these genes would be under rapid evolution^103^.

We also detected individual sites under positive selection for the ancestral branch of web spiders using 5,680 orthologous genes. A mixed-effects maximum likelihood approach (MEME) was employed by using the MEME framework^104^ in Hyphy v2.5.25 (http://www.hyphy.org/)^105^ with default parameters. MEME allows the distribution of ω to vary from site to site (the fixed effect) and also from branch to branch at a site^103^. This programme showed the significance of the episodic positive selection for each site using a likelihood ratio test.

### Differential expression analyses of spider brains

To identify differential expressed genes across burrowing and web-building species, the brain transcriptome samples of two web-building spiders, *Hylyphantes graminicola* and *Parasteatoda tepidariorum*, and two burrowing spiders, *Atypus karschi* and *Luthela Beijing*, were mapped to their trimmed-orthologues. For each species, the expression level matrix of orthologues was constructed using RSEM v1.3.1 (ref. ^106^). We performed differential expression analysis using edgeR package v3.26.8 (ref. ^107^) with a FDR of 0.05 and a fold change of 4.

### Linking molecular evolution and neuron innovation

We next focused on the expanding gene family and PSGs/REGs that are specifically expressed in the spider brain. We calculated the average expression level and percent of expression for each cell cluster using the *AverageExpression* function (idents = ‘cell.type’). Using DotPlot function (pct.exp > 25, avg.exp > 2) in Seurat, we manually checked gene expression patterns for each gene and identified the major cell type that expressed the candidate genes. The potential functions of expanding gene family/PSGs in each cluster were examined by GO enrichment.

### Sex-biased expression and sex-specialized cell types

For the sex-bias analysis, we first obtained the gene expression matrix using the AverageExpression function (idents = ‘sex’) in Seurat. Next, we found the sex-bias genes using *FindMarkers* function for all sample pairs (for example, find the different expression gene between male_1 and female_1 using the parameters (min.pct = 0.25, ident.1 =‘male_1’, ident.2 = ‘female_1’, only.pos = F) based on the non-parametric Wilcoxon rank sum test. Only genes that showed significant expression differences in all comparisons were considered as sex-bias genes. We searched for sex-biased genes for each cell using the same strategy. We then applied the UCell package v2.2 (ref. ^108^) to evaluate the UCell signature score of male-biased and female-biased genes for each cell using the *AddModuleScore_UCell* function. The UCell score is calculated as the difference between the average expression of the genes in the module score and the genes in the background for each cell. Scores close to zero indicate a similar expression, positive scores indicate higher expression and negative scores indicate lower expression of the genes in the gene set than the background genes. Gene scores for each cluster were calculated using AverageExpression (features = signature.names**)** and visualized using dotplot by ggplot2 package v3.3.5 (ref. ^109^).

### Measurement of monoamine transmitters

The octopamine (OA) and norepinephrine (NE) concentrations were measured via reversed phase UltiMate High Performance Liquid Chromatography with electrochemical detection (UHPLC-ECD, DIONEX UltiMate 3000, RS Pump)^110^.

### Immunostaining of spider brains

Immunostaining was conducted following similar procedures for *Drosophila* adult brain staining^111^. Briefly, spiders were dissected in 0.015% PBST (phosphate-buffered saline with 0.015% Triton X-100) and fixed with 4% PFA at room temperature on a shaker for 20 min. Samples were then washed with 0.5% PBST for 4 × 15 min. After washing, samples were blocked by 5% goat serum in 0.5% PBST at room temperature on a shaker for 30 min. The primary antibody and its dilution ratio were adopted from previous publications^112,113^. Mouse anti-SYNORF1 and anti-Fasciclin II antibody was purchased from Developmental Studies Hybridoma Bank and diluted to 1:1,000 in the blocking buffer. Samples were treated with the primary antibody for 48 h and then washed for 4 × 15 min. The secondary antibody Alexa Fluor 488 anti-Mouse (Invitrogen A11001) was used at 1:250, and samples were incubated for 72 h followed by similar washing procedures. Rabbit anti-norepinephrine (NE) antibody (immusmol, IS1042) with STAINperfect immunostaining kit A (SP-A-1000) was used for NE immunolabeling. An Olympus FV1000 microscope with a 20× air lens (NA = 0.8) was used for confocal imaging.

### RNA in situ localization

Spider cRNA probes were prepared using T7 promoter sequence (Supplementary Table 10). In vitro transcription of DNA template was carried out using thermocycler followed by RNA probe synthesis using DIG RNA labelling kit using manufactures protocol (Roche Diagnostics). In brief, spider brains were fixed in 4% paraformaldehyde in for 20–60 min. The brains were washed using 1× PBS with 0.1% Tween 20 (PBST). Then it was permeabilized using proteinase K (1 μg ml^−1^) and fixed again with 4% PFA. Samples were incubated in hybridization solution at 50 °C for 1 h. A DIG-labelled cRNA probe was used and heat-denatured by incubating it for 5 min at 80 °C. The brains were then washed using wash buffer and were blocked with 5% BSA blocking solution at RT for 1.5 h followed by incubation with anti-Digoxigenin-FITC antibody (21H8) (FITC) (ab119349) (1:500) at 4 °C for overnight. Brain samples were washed four times for 15 min with DIG wash buffer (Roche Diagnostics) and ready for confocal imaging.

### RNAi experiment

#### Candidate gene

Gene *ben* is both a positively selected gene and a rapidly evolving gene, and it is highly expressed in mushroom body-like neurons. The expression levels were similar across different developmental stages. The dsGFP was synthesized and used as a control.

#### Experimental subject

Wild-type adult spiders were obtained from the greenhouse in the Institute of Zoology, Chinese Academy of Sciences. The spiders were then housed individually in a plastic box (50 mm × 50 mm × 40 mm) at temperature- and humidity-controlled conditions (24–26 °C and 50–60% humidity) in a 14 h/10 h light–dark cycle. Offspring (subadult females) of wild spiders were used for experiments. Experimental subjects were caged individually from second instar juveniles to subadults. The sample size of each measured behaviour and fitness trait is shown in Supplementary Table 8.

#### RNAi in vivo

For RNAi, 495 base pair fragments of *ben* were PCR amplified from cDNA. The T7 promoter sequence attached primers were used for PCR amplification of the double-stranded RNA (dsRNA) templates using HiScribe T7 (NEB) according to the manufacturer’s instructions. Synthesized dsRNA was quantified with a Nanodrop 2000 (Thermo Fisher). The final concentration of double-stranded RNA used for injection was 5µg µl^−1^. Before injection, the spiders were anaesthetized with CO_2_. A CellTram air microinjector (Eppendorf) was used for injection.

#### Validation of knockdown

The knockdown effects were validated with RT-qPCR after 24 h upon dsRNA injection using three technical replicates per gene per sample. The whole body of spiders was collected, and total RNA was extracted using RNAiso Plus (Takara) according to the manufacturer’s instructions. Reverse transcription was performed by using HiScript III RT SuperMix for qPCR (+gDNA wiper) (Vazyme Biotech). Quantitative real-time PCR was performed using Taq Pro Universal SYBR qPCR Master Mix (Applied Biosystems). Glyceraldehyde-3-phosphate dehydrogenase (GAPDH) gene was used as a reference housekeeping gene, as past studies have shown it has relatively similar expression across all tissues^114^. The primers for qPCR were listed in Supplementary Table 10.

#### Behavioural assay

We tested the effects of the expression of the candidate gene (*ben*) on spider web-building and predatory behaviour. We used four indicators to test behavioural change after RNAi: in the first experiment, the spiders usually stay in the hub of their web. We removed all silk/webs in the plastic box after 24 h upon dsRNA injection and observed whether they would build webs in the same position. Using an in-house Python script-based method (Supplementary Code), the length of the SSt (supporting structure, a silken line) and the number of GF (Gumfooted lines, threads with viscid basal portions) were also used to quantify the web quality. In our second experiment, we dropped a fruit fly on the spider’s web and allowed the spider to perform normal prey capture behaviour with each prey item (locate prey, extract it from the web, wrap it in silk, secure it to the hub) and eat. If the fly broke free from the web within 10 min, we dropped another fruit fly on the web. After 10 min, we removed all the live fruit flies. We fed the spider once per day and recorded how often the spider captured the prey each day.

### Statistical analyses

Kruskal Wallis test with Dunn’s multiple-comparison post-hoc test was used to compare groups of data. Non-parametric two-tailed Mann–Whitney U tests were used to compare two distributions. All measurements were taken from independent samples. All graphs and statistical analyses were generated using GraphPad Prism software unless otherwise stated.

### Reporting summary

Further information on research design is available in the Nature Portfolio Reporting Summary linked to this article.

### Supplementary information

Supplementary Information Supplementary Figs. 1–9 and descriptions for Supplementary Tables 1–10, Data 1–20 and Code.

Reporting Summary

Supplementary Tables 1–10 Supplementary Tables 1–10.

Supplementary Data 1–19 Supplementary Data 1–19.

Supplementary Data 20 Predicted precursors of neuropeptides in *Hylyphantes graminicola*.

Supplementary Code 1–10 Supplementary code.

### Source data

Source Data Fig. 2 Statistical source data.

Source Data Fig. 3 Statistical source data.

Source Data Fig. 4 Statistical source data.

Source Data Fig. 5 Statistical source data.

Source Data Fig. 6 Statistical source data.

Source Data Extended Data Fig. 1 Statistical source data.

Source Data Extended Data Fig. 4 Statistical source data.

Source Data Extended Data Fig. 5 Statistical source data.

Source Data Extended Data Fig. 6 Statistical source data.

Source Data Extended Data Fig. 7 Statistical source data.

## Data Availability

The raw and processed data of single-cell transcriptomes of spider brains are deposited into the GEO database (with accession code GSE241696); all raw transcriptome data have been deposited into the NCBI Sequence Read Archive (SRA) database with a BioProject accession PRJNA934409 and a BioSample accession SAMN33275591–SAMN33275618 and SAMN36403531–SAMN36403537. Raw DNA sequencing data of *Luthela Beijing* and *Atypus karschi* are deposited into the Genbank with BioProject accession: PRJNA1008782 and PRJNA1010389. The genome assemblies of *Luthela Beijing* and *Atypus karschi* were available in Science Data Bank: 31253.11.sciencedb.07403. The functional annotations of protein-coding genes, metadata, results from genetic analysis and GO lists and other source and processed data are available in the Supplementary Data. Source data are provided with this paper.

## Code availability

Data analysis scripts and code are available via Figshare (10.6084/m9.figshare.22303228)^115^.
