# Supplementary material for: Single-cell transcriptomics reveals the brain evolution of web-building spiders
Source: Nat Ecol Evol. 2023 Nov 2;7(12):2125–42. doi: 10.1038/s41559-023-02238-y (PMC10697844; doi:10.1038/s41559-023-02238-y)
Supplement: Supplementary file 1 — Supplementary Figs. 1–9 and descriptions for Supplementary Tables 1–10, Data 1–20 and Code. [file 41559_2023_2238_MOESM1_ESM.pdf]

nature ecology & evolution

Article https://doi.org/10.1038/s41559-023-02238-y

Single-cell transcriptomics reveals the brain evolution of web-building spiders

In the format provided by the authors and unedited

Supplementary Figure 1 Norepinephrine immunostaining in spider brain.

Supplementary Figure 2 Mushroom body staining in spider brain.

Supplementary Figure 3 Expression patterns of marker genes related to GABA neurons (*Gad*), cholinergic neurons (*ChAT*) and glutamatergic neurons (*VGlut*) in brain scRNA-seq samples of *Hylyphantes graminicola*.

Supplementary Figure 4 Dotplot showing single-cell expression level of expanded gene families in different spiders.

Supplementary Figure 5 The RNA contamination level (up) and doublet cell proportion (down) for monoaminergic neuron clusters.

Supplementary Figure 6 The doublet cell proportion (up) and RNA contamination level (down) for all cell clusters.

Supplementary Figure 7 Cell communication pattern at different resolutions.

Supplementary Figure 8 Pairwise transcriptional similarity of cell clusters from *Drosophila* and *Hylyphantes* by SAMap program.

Supplementary Figure 9 Gene networks between transcription factors (TFs) and neuropeptides.

Supplementary Table 1 Sequencing and mapping statistics, including numbers of cells, mean reads per cell, and number of genes detected per cell.

Supplementary Table 2 Seurat object metadata for all cells after stringent quality control. RNA_snn_res.2 was used for the final analysis.

Supplementary Table 3 Markers used for cell annotation

Supplementary Table 4 Marker gene lists.

Supplementary Table 5 Assembly statistics and BUSCO assessment of two assembled genomes.

Supplementary Table 6 Positive selection genes (PSGs) or rapid evolution genes (REGs) at common ancestor of aerial web-building spiders (node c).

Supplementary Table 7 GO analysis of positive selection genes (PSGs) or rapid evolution genes (REGs) at common ancestor of aerial web-building spiders (node c).

Supplementary Table 8 Samples used for RNAi experiment and qPCR.

Supplementary Table 9 Omics data used in this study.

Supplementary Table 10 qPCR and dsRNA and RNA-FISH primers in study.

Supplementary Data 1-20

Supplementary Code 1-10

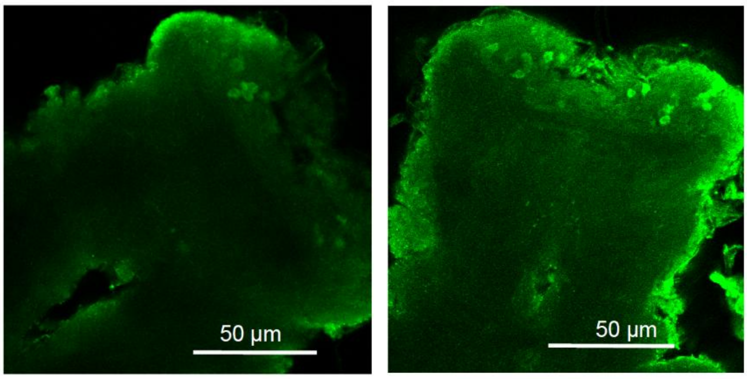

Supplementary Figure 1 Norepinephrine immunostaining in spider brain. Anti-Noradrenaline (NE) antibody (IS1042) together with the STAINperfect immunostaining kit A (SP-A-1000) were used. Four independent biological replicates were performed to confirm the distribution of Norepinephrine neurons.

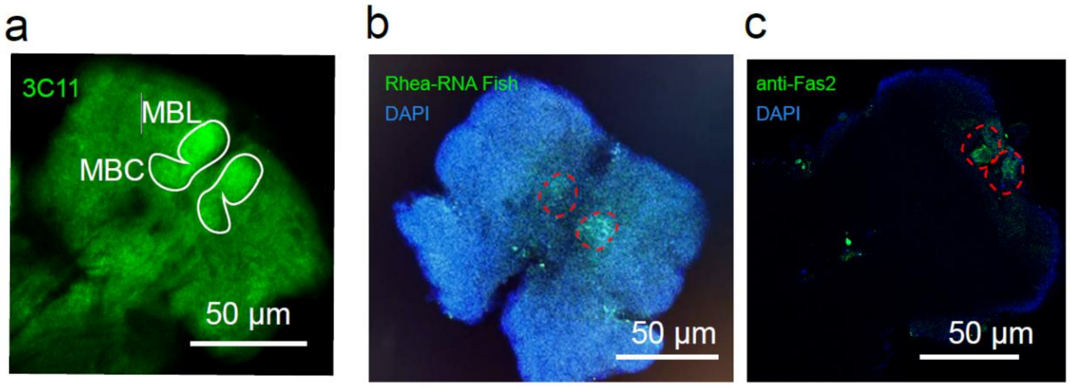

Supplementary Figure 2 Mushroom body staining in spider brain. a, 3C11 staining showed the structure of the mushroom body (MB) in the spider brain. **b,** RNA fluorescence in situ hybridization (FISH) for *rhea* marks subset of MBs. **c,** anti-Fas2 immune-staining showed the expression pattern of Fas2 (green). Two independent biological replicates were performed to confirm the expression of *rhea* and *Fas2* in the brain. Three independent biological replicates of 3C11 staining were performed to confirm the brain structure.

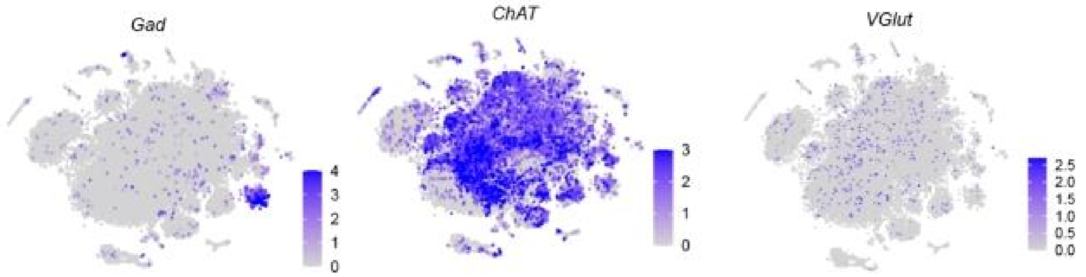

Supplementary Figure 3 Expression patterns of marker genes related to GABA neurons (*Gad*), cholinergic neurons (*ChAT*) and glutamatergic neurons (*VGlut*) in brain scRNA-seq samples of *Hylyphantes graminicola*.

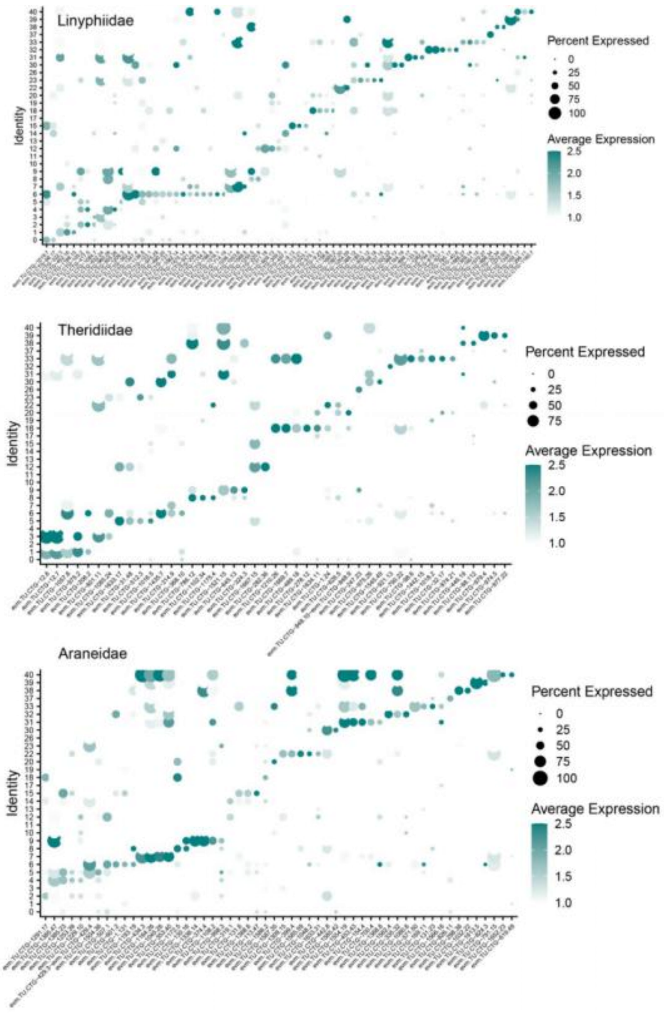

Supplementary Figure 4 Dotplot showing single-cell expression level of expanded gene families in different spiders. We presented the expanded gene families for Linyphiidae (up), Theridiidae (middle), and Araneidae (down) compared to other arthropod species in Fig. 5 of the main text. The corresponding homologous genes were obtained from the genome of the *Hylyphantes graminicola.*

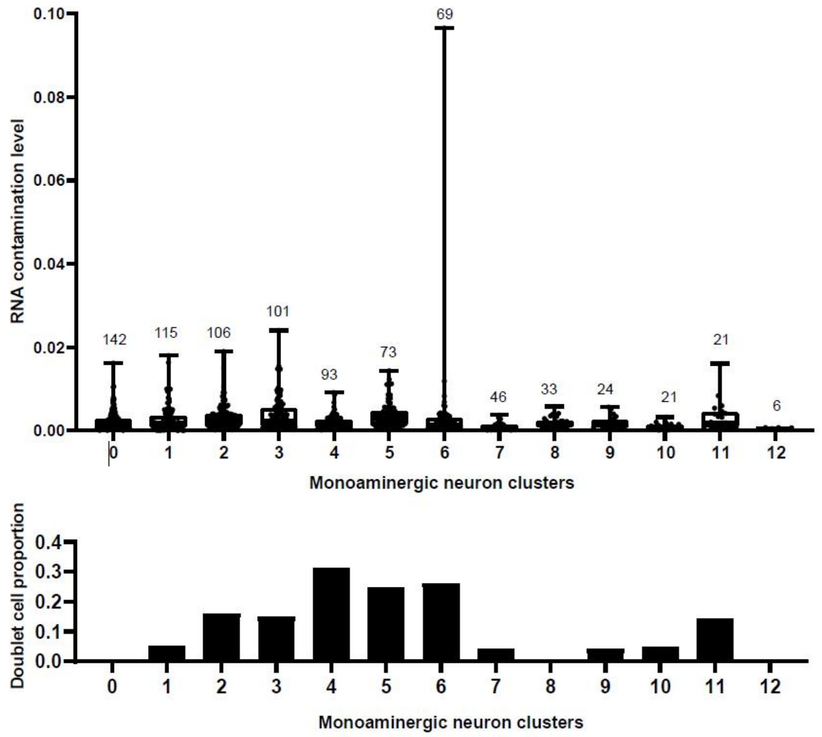

Supplementary Figure 5 The RNA contamination level (up) and doublet cell proportion (down) for monoaminergic neuron clusters. The number above the box indicates the cell number of each cluster. Box plots show minimum to maximum (whiskers), 25–75% (box), and median (band inside) with all data points.

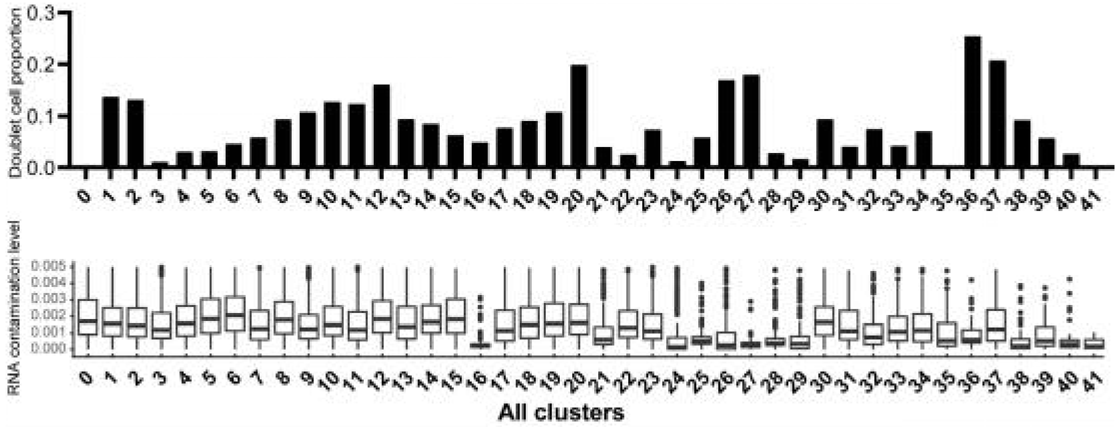

Supplementary Figure 6 The doublet cell proportion (up) and RNA contamination level (down) for all cell clusters. Box plots show minimum to maximum (whiskers), 25–75% (box), median (band inside) with data points less than 0.005. The cell number of each cluster ranged from 28 (cluster 41) to 3233 (cluster 0).

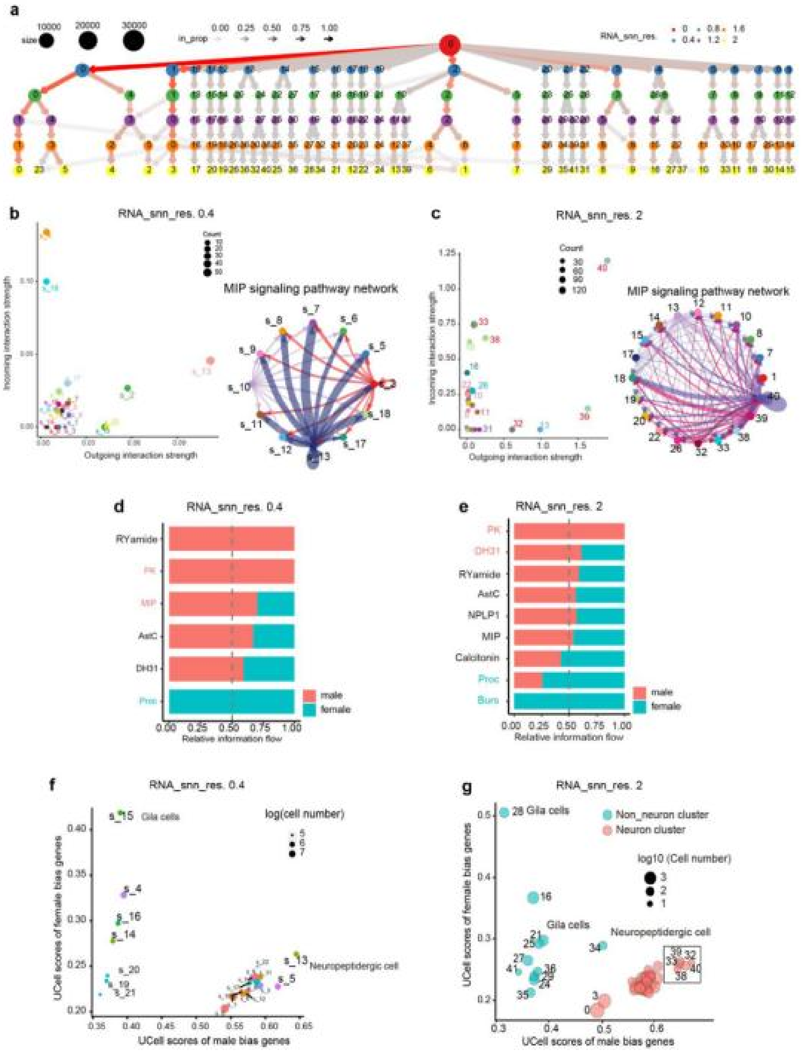

Supplementary Figure 7 Cell communication pattern at different resolutions. a, Comparison of different cluster resolutions. **b-c,** The outgoing and incoming interaction strength for each cell inferred by CellChat. The right panel shows the communication networks of *MIP* signaling pathway network, which is the dominant neuropeptide signal. **d-e,** Comparison of the overall information flow of each signaling pathway between males and females. Significantly different signaling pathways were colored red (male) and blue (female). **f-g,** Ucell scores of male bias genes and female bias genes. **b, d, f,** Related results at resolution 0.4; **c, e, g,** Related results at resolution 2.

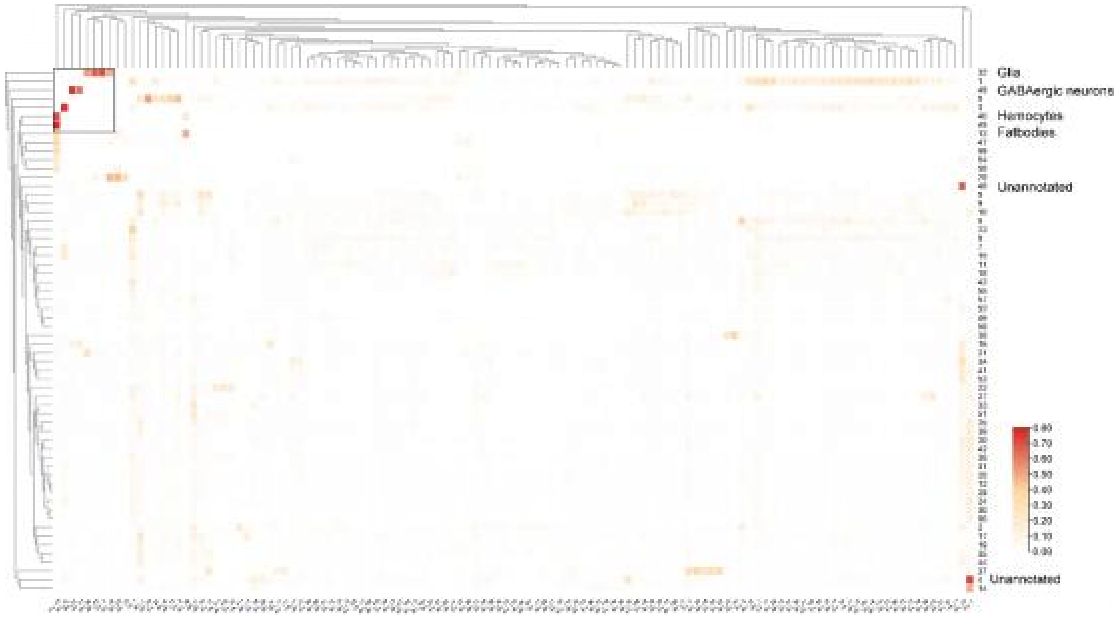

Supplementary Figure 8 Pairwise transcriptional similarity of cell clusters from *Drosophila* and *Hylyphantes* by SAMap program. Red squares in black box indicate the conserved cell cluster types between brain scRNA-seq samples of *Drosophila* and *Hylyphantes.*

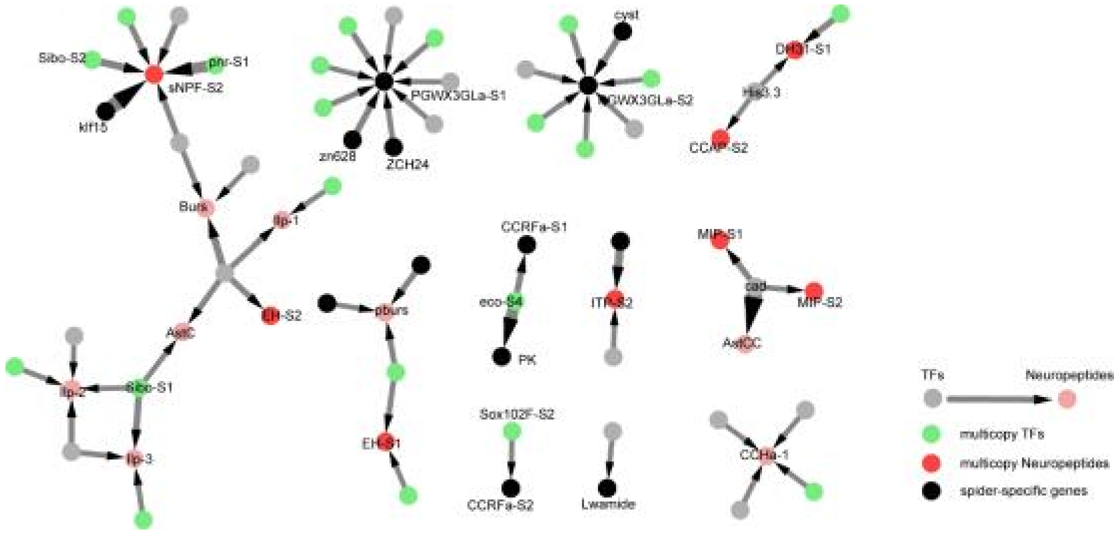

Supplementary Figure 9 Gene networks between transcription factors (TFs) and neuropeptides. Grey and green dot represent TFs and multicopy TFs, respectively; pink and red dots represent neuropeptides and multicopy neuropeptides respectively; black dots represent the specific genes of spiders. Line thicknesses indicate the interaction strengths.
