# Supplementary material for: Single-cell transcriptomics reveals the brain evolution of web-building spiders
Source: Nat Ecol Evol. 2023 Nov 2;7(12):2125–42. doi: 10.1038/s41559-023-02238-y (PMC10697844; doi:10.1038/s41559-023-02238-y)
Supplement: Supplementary file 2 — Reporting Summary [file 41559_2023_2238_MOESM2_ESM.pdf]

nature portfolio

Corresponding author(s): Shuqiang Li

Last updated by author(s): Sep 11, 2023

Reporting Summary

Nature Portfolio wishes to improve the reproducibility of the work that we publish. This form provides structure for consistency and transparency in reporting. For further information on Nature Portfolio policies, see our Editorial Policies and the Editorial Policy Checklist.

Statistics

For all statistical analyses, confirm that the following items are present in the figure legend, table legend, main text, or Methods section.

n/a	Confirmed	
☐	☒	The exact sample size (n) for each experimental group/condition, given as a discrete number and unit of measurement
☐	☒	A statement on whether measurements were taken from distinct samples or whether the same sample was measured repeatedly
☐	☒	The statistical test(s) used AND whether they are one- or two-sided<br>*Only common tests should be described solely by name; describe more complex techniques in the Methods section.*
☒	☐	A description of all covariates tested
☐	☒	A description of any assumptions or corrections, such as tests of normality and adjustment for multiple comparisons
☐	☒	A full description of the statistical parameters including central tendency (e.g. means) or other basic estimates (e.g. regression coefficient) AND variation (e.g. standard deviation) or associated estimates of uncertainty (e.g. confidence intervals)
☐	☒	For null hypothesis testing, the test statistic (e.g. F, t, r) with confidence intervals, effect sizes, degrees of freedom and P value noted<br>*Give P values as exact values whenever suitable.*
☒	☐	For Bayesian analysis, information on the choice of priors and Markov chain Monte Carlo settings
☒	☐	For hierarchical and complex designs, identification of the appropriate level for tests and full reporting of outcomes
☐	☒	Estimates of effect sizes (e.g. Cohen's d, Pearson's r), indicating how they were calculated

Our web collection on statistics for biologists contains articles on many of the points above.

Software and code

Policy information about availability of computer code

Data collection	No software was used.
Data analysis	Softwares used to analyze the data were described in details in the Methods section of the manuscript and listed below: CellRanger V4.0.0, Seurat v4.0, Harmony v0.10, clustree v0.5.0, BLAST v2.10.0, OrthoFinder v.2.3.118, Diamond v0.8.22, SignalP v6.0b, DeepTfactor, ClusterProfiler v3.18.1, REViGO, CellChat v1.1.3, GENIE3 v3.16, Cytoscape v3.7.2, wgdi v0.1.6, YN00 v4.9, TBtools v0.665, MAFFT v7.455, R v4.0, wtdbg2 v2.5, Racon v1.4.17, NextPolish v1.4.0, braker-2.1.6, Augustus v3.3, GenomeThreader v1.7.3, STAR v2.7.3a, transdecoder v5.5.0, EVidenceModeler v1.1.1, IQTREE v1.6.12, CAFÉ v5.0, PAML v4.9j, trimAL v1.4.rev15, Hyphy v2.5.25

For manuscripts utilizing custom algorithms or software that are central to the research but not yet described in published literature, software must be made available to editors and reviewers. We strongly encourage code deposition in a community repository (e.g. GitHub). See the Nature Portfolio guidelines for submitting code & software for further information.

Data

Policy information about availability of data

All manuscripts must include a data availability statement. This statement should provide the following information, where applicable:

- Accession codes, unique identifiers, or web links for publicly available datasets
- A description of any restrictions on data availability
- For clinical datasets or third party data, please ensure that the statement adheres to our policy

The Raw and processed data of single-cell transcriptomes of spider brain are deposited into the GEO database (with accession code GSE241696); All raw transcriptome data have been deposited into the NCBI Sequence Read Archive (SRA) database with a BioProject accession PRJNA934409 and a BioSample accession SAMN33275591- SAMN33275618 and SAMN36403531-SAMN36403537. Raw DNA sequencing data of Luthela Beijing and Atypus karschi are deposited into the Genbank with BioProject accession: PRJNA1008782 and PRJNA1010389. The genome assemblies of Luthela Beijing and Atypus karschi were available in Science Data Bank: 31253.11.sciencedb.07403. The functional annotations of protein-coding genes, metadata, results from genetic analysis and GO lists and other source and processed data are available in supplementary data.

Human research participants

Policy information about studies involving human research participants and Sex and Gender in Research.

Reporting on sex and gender	*Use the terms sex (biological attribute) and gender (shaped by social and cultural circumstances) carefully in order to avoid confusing both terms. Indicate if findings apply to only one sex or gender; describe whether sex and gender were considered in study design whether sex and/or gender was determined based on self-reporting or assigned and methods used. Provide in the source data disaggregated sex and gender data where this information has been collected, and consent has been obtained for sharing of individual-level data; provide overall numbers in this Reporting Summary. Please state if this information has not been collected. Report sex- and gender-based analyses where performed, justify reasons for lack of sex- and gender-based analysis.*
Population characteristics	*Describe the covariate-relevant population characteristics of the human research participants (e.g. age, genotypic information, past and current diagnosis and treatment categories). If you filled out the behavioural & social sciences study design questions and have nothing to add here, write "See above."*
Recruitment	*Describe how participants were recruited. Outline any potential self-selection bias or other biases that may be present and how these are likely to impact results.*
Ethics oversight	*Identify the organization(s) that approved the study protocol.*

Note that full information on the approval of the study protocol must also be provided in the manuscript.

Field-specific reporting

Please select the one below that is the best fit for your research. If you are not sure, read the appropriate sections before making your selection.

☐ Life sciences ☐ Behavioural & social sciences ☒ Ecological, evolutionary & environmental sciences

For a reference copy of the document with all sections, see nature.com/documents/nr-reporting-summary-flat.pdf

Ecological, evolutionary & environmental sciences study design

All studies must disclose on these points even when the disclosure is negative.

Study description	This study is aimed to determine the spider's neuron type and explore how gene selection and gene family evolution shape neuron specificity and behavior diversity. We set 5 replicates for spider brain single-cell transcriptomic sequencing.Two spiders were selected for genome sequencing and 14 species were used for comparative genomic analysis.
Research sample	Spider brain tissue of Male and female from Hylyphantes graminicola; adult purseweb spider and segmented spider for genome sequencing.
Sampling strategy	We obtained a total of 40,233 single cells from the five replicates. This is around 0.5-1.2 times the estimated cell number of 30,000 (e.g. Argiope aurantia) –100,000 (e.g. Cupiennius salei) in a single individual spider brain, and thus is expected to be sufficient for capturing most cell types in the spiders brain. Two spiders were selected for genome sequencing because they represent the plesiomorphic burrowing spiders. Fourteen genomes covered the major lineages of Arachnida.
Data collection	P.Y.J. and B.Y.Z. prepared samples for 10X single-cell RNA-seq. Single-cell transcriptomics amplification and library preparation were performed at Capitalbio Technology Corporation (Beijing, China). Y.M.Z. and Y.X.S. prepared samples for genome sequencing. The

	high-fidelity (HiFi) libraries were sequenced on the PacBio Sequel II system in Circular Consensus Sequencing (CCS) mode at Novogene technology co., LTD (Beijing, China).
Timing and spatial scale	Sample collection between 2020-2021.
Data exclusions	N/A
Reproducibility	Information about the single-cell transcriptomic and comparative genomics methods used in this paper are included in our material and methods.
Randomization	N/A
Blinding	N/A

Did the study involve field work? ☐ Yes ☒ No

Reporting for specific materials, systems and methods

We require information from authors about some types of materials, experimental systems and methods used in many studies. Here, indicate whether each material, system or method listed is relevant to your study. If you are not sure if a list item applies to your research, read the appropriate section before selecting a response.

Materials & experimental systems

n/a	Involved in the study	
☐	☒	Antibodies
☒	☐	Eukaryotic cell lines
☒	☐	Palaeontology and archaeology
☐	☒	Animals and other organisms
☒	☐	Clinical data
☒	☐	Dual use research of concern

Methods

n/a	Involved in the study	
☒	☐	ChIP-seq
☒	☐	Flow cytometry
☒	☐	MRI-based neuroimaging

Antibodies

Antibodies used	Mouse anti-SYNORF1, Alexa Fluor 488 anti-Mouse, Rabbit anti-norepinephrine (NE) antibody , Mouse anti-Fasciclin II.
Validation	Mouse anti-SYNORF1 and its dilution ratio were validatied from previous publications (Brenneis et al. 2022; Steinhoff et al. 2017).<br>Anti-norepinephrine was validatied from previous publication (Robertson et. al. 2013)<br>Mouse anti-Fasciclin II was validatied from previous publication (Klämbt 2000)

Animals and other research organisms

Policy information about studies involving animals; ARRIVE guidelines recommended for reporting animal research, and Sex and Gender in Research

Laboratory animals	First generation (G1) of wild collected spiders were used for Immunostaining, RNAi experiments and RNA-seq from different tissues.
Wild animals	Wild-collected spiders of aerial web-building spider for brain dissection were collected from Anci District, Langfang City, Hebei Province, China. Sample of the purseweb spider was collected from the bamboo forest Tongji Town, Chengdu City, Sichuan Province, China (31.18°N, 103.84°E). Sample of the segmented spider was collected from Purple Bamboo Park, Haidian District, Beijing City, China (39.94°N, 116.32°E).
Reporting on sex	Single-cell libraries from two male and three female samples were used to compare sexual difference in cell type and cell-cell communication patterns.
Field-collected samples	Wild-collected spiders were maintained individually in a glass tube or plastic box at temperature- and humidity-controlled condition (24–26 ℃ and 50–60% humidity) in a 14hr/10hr light-dark cycle in Institute of Zoology, Chinese Academy of Sciences.
Ethics oversight	No ethical approval was needed because spiders used in this study are common species with huge population size in the field and are not threatened species.

Note that full information on the approval of the study protocol must also be provided in the manuscript.
