# Supplementary material for: Single-cell transcriptomics reveals the brain evolution of web-building spiders
Source: Nat Ecol Evol. 2023 Nov 2;7(12):2125–42. doi: 10.1038/s41559-023-02238-y (PMC10697844; doi:10.1038/s41559-023-02238-y)
Supplement: Supplementary file 5 — Predicted precursors of neuropeptides in Hylyphantes graminicola. [file 41559_2023_2238_MOESM5_ESM.doc]

Supplementary Data20: Predicted precursors of neuropeptides in *Hylyphantes graminicola*

Blue indicates the predicted signal peptide by SignalP 6.0. Grey means convertase cleavage sites and amino acid residues likely to be removed by carboxypeptidase. Pink represents Amidation cleavage sites. Yellow marks the conserved cysteine residues in a neuropeptide.

**Achatin**

>evm.model.CTG_47.16

MAHLQKPVIIVFLLLGLKLASCLADDVDSSDARELFYRSSLGFQKESRQLPHLHHTSDEESPEDAEETPNGSHQFEKRGFGEKRGFGEKRGFGEKRGFGEKRGFGEKRGFGEKRGFGEKRGFGEKRGFGEKRGFGEKRGFGEKRGFGEKRGFGEKRSYISKDSNNYVSFITQWSHILKPEELNRFRAEHILPYSEQYHG

**Agatoxin-like peptide (ALP)**

>evm.model.CTG_1553.3

MQPLLWTALSLLLLTVLLDAAVPPDALERNIPEDYSDVNLESFLGKQYKRSGCVRRGGSCDGKPNDCCPNSACRCNLWGTNCRCERQGLLQQWGRRRK

>evm.model.CTG_569.27

MQWELESDISSTALAWQISPFSTESWDVNERRTSTERVASCLCTVRDTPVYLCLRIRGARACEGLLRVYKGAPREAPTQLSSPASDTRHLSLSSSSSCLKPCPCSAMQTQLLSSAVLVLLLVQVALAIPPALDRNLPEDYSENALEQLLGRSDKRGCIQRGGGCDARPNDCCPNSACRCNLWGTNCRCDRQGLFQQWGRRK

**Allatostatin B (AstB)**

>evm.model.CTG_578.5

MFSPKMHRISLTLLCILPLIYFASCNPGLPDEPPKPDANPHASDLENLHGQLDTSLVSEEEDDPEKRGWERLSNVWGKRADWNKLNNMWGKRGDWNKLNNMWGKRAAEWNKLNNMWGKRGDGNWNKLNNMWGKRGDGDWNKLNNMWGKRGDGDWNKLNNMWGKRAAEWNKLNNMWGKRAEWNKLNNMWGKRAEWNKLNNMWGKRGSSREDGSKRTQWNNLKSVWGKRDAGWEGPVAWDTNEPNEDSFQGVYVPDQM

**Allatostatin B (AstB)-mip**

>evm.model.CTG_1394.2

MFSPKMHRISLTLLCILPLIYFASCNPGLPDEPPKPDANPHASDLENLHGQLDTSLVSEEEDDPEKRGWERLSNVWGKRADWNKLNNMWGKRGDWNKLNNMWGKRAAEWNKLNNMWGKRGDGNWNKLNNMWGKRGDGDWNKLNNMWGKRLDGDWNKLNNMWGKRAAEWNKLNNMWGKRAEWNKLNNMWGKRAEWNKLNNMWGKRGSSREDGSKRTQWNNLKSVWGKRDAGWEGPVAWDTNEPNEDSFQGVYVPDQM

**Allatotropin (AT)**

>evm.model.CTG_216.1

MSRLLLLSVFVVAVVCVHSSSSSSSQIRQKRGFRNAALSTARGFGKRTQQDTLLEEALARPVASSWLAEQMARNPVLARLFVDKMVDQNGDGMIQPEEMYPTV

**AstC**

>evm.model.CTG_1075.4

MEPTRVLLLLLLLLGVSGAAPPPPSSFLMDAPPWDEEDATPQQVAPFPAKRQTTRYHQCYFNPISCFK

**AstCC**

>evm.model.CTG_63.18

MGGRPLKTGGGPRDVATSNMEIAAPKFHLLLLAACLLVNTAQASQQESSPLLRNKRPTVLLNRLLSTLQTMLGGEEAPLPPPAPQVPLHPPPHHQELERRGGFPRHYWKCYFNAVSCFRRKRHTLQSVSQ

**AstCCC**

>evm.model.CTG_1140.24

MRLRIVSVKRPGNDLPDADSSSNSAITWVLLGSGLHHSSRMVYYMRICSLLVASLLLVSFTSAKAMPQQEKLYFPSQLDVTDDDGSPDNALLNYLFARQMVRRLRSNMDVSDLQRKRSYWKQCAFNAVSCFGKK

**Bursicon(Bursα)**

>evm.model.CTG_160.9

MSLASLVACCLLQATLWSLSEGRGEVCHLRPVIHVLQQPGCIPKPIPSFACQGSCSSYVQVSGSKFWQVERSCMCCQEMGEREASIGIFCPKASPRFRKIVTRAPVECMCRPCTAVDEASVHPQEFSPMLTAPKELLLKDMPQPPEAYHIRGEFGPDKEIPVV

**Bursicon(Bursβ)**

>evm.model.CTG_160.10

MPSNPKKPSLIWWWSILTLSLAYCREHNEQDEELAQVVAKGSEAVCETLPSTIQVAKEEYDDLGNVVRSCEGTIGVTKCEGTCNSQIQPSVVTPTGFLKECHCCRETWMRQREILLTDCYTQDGKRIFGQQGTMVIHLKEPEACACHKCGL

**Calcitonin-like (Cal)**

>evm.model.CTG_1293.44

MATDSFHAYLALFGILLLATIAHSQDLAFEDNIKELLSKRKNLDMVRQMVEDVDNQLNRLHKVSCMLRMPGLDCSSYALDDRVKDLLVSGSKKRSFRSHQQLLQQPLYSCDFKVGNPEGCLGKLIEADNKDNSHWGDPDGPGKK

>evm.model.CTG_508.11

MATDSFHAYLALFGILLLATIAHSQDLAFEDNIKELLSKRKNLDMVRQMVEDVDNQLNRLHKVSCMLRMPGLDCSSYALDDRVKELLVGGSKKRSFRSHQQLLQQPLYSCDFKVGNPEGCLGKLIEADNKDNSHWGDPDGPGKK

>evm.model.CTG_571.10

MKMENSLVFILSLLVLAEVADLSFSSPIIPQRSLRDIVYKRRQLSYIKNLLQDLDDKLEKLEKRSCAINIPGSDCDYSEISGTGKDQGFWNSRLVPGKRLRYRRNLENVF

**CCAP**

>evm.model.CTG_40.6

MLKEHMFVLLLLCVLSVWGEIRQESRLQKRVFCNAFTGCGGMSSRTKKFGKRSLREINPIEYERRAAKIPILYPELMQILLETKTFPQDKKEIYFDTIDSGPSISIDKERLSRNV

**CCHamide (CCHa)**

>evm.model.CTG_1026.2

MTKRTVVAFALGTVIFGIHILNACSYSAGHCKQYGHACLGGHGKRSRTESNSPLMDLLLSDVLKNQGKVSPQLSDLDRRNLQDFWETAFNKNDEEWISDGDERYNRPIFN

**CCHamide (CCHa)**

>evm.model.CTG_1183.3

MLDNRYILLIAAVIAVIEIFGTAYAGGSCQSYGHSCLGGHGKRNSETPYALHALLQRAMRTRNIVPFNDYRFPDSNTYDFLKGFTDGIKELEDSAEPQK

**CCRFamide (CCRFa)**

>evm.model.CTG_571.11

MSLHPIFLFSLTVMVWLWCTTEASRFSRNEARCAVVCMNKDSEECRMCNTRIPMRFGKRNNRAMPAPAGYLYDLDKMEKPQPDVRFLALVEGVDGSDLDSDEK

>evm.model.CTG_674.29

MKSLVFTFAMTALMLYLIPESSAELLKRCESVCSRSDGQDFCQRCRMRVPMRFGKRVVPTSDVTIQKKGMRQLAGEAEEEDNNSGYDDSGSQESGSNANVREEARFLLDSMKNFARDLENWNNEVYDQ

**Corazonin**

>evm.model.CTG_40.5

MVAINMNSTLTLLLLLQIAMLVVGQTYQYSRGWTNGKKRSLSSERDNEQFPIKYSLDGSKDGEEQPSLDGLMLVNLKYSKFLEGTGQTTEQVVNDELTENFKSIHIPSSKVSEKYFRKWLYKLLTKLDKQDKVPQK

**Crustacean cardio-active peptide (CCAP)**

>evm.model.CTG_208.1

MKINQCRRLDITMVNSTYLTFIMLTVLAIVFTVSSDSLDQDRLDFRVLQKRPFCNAFTGCGRKRSGESGGAAASNKLYQLLQQRMAEVEALKGLLDEVAHYCVIDVDRFG

>evm.model.CTG_246.26

MVNSTYLTFIMLTVLAIVFTVSSDSLDQDRLDFRVLQKRPFCNAFTGCGRKRSGESGGAAASNKLYQLLQQRMAEVEALKGLLDETRK

**Crustacean hyperglycemic hormone/Ion transport peptide-like (CHH/ITP)**

>evm.model.CTG_539.1

MYQKIMVSVGCVVWLMAAGVGCQDMWEESHKSSGCLGIYDRYNMAHLERVCDECFELYKEQAIHKSCRSHCFGSKTFFDCMEAVALDGFRKEAVLLSWESLRSIK

>evm.model.CTG_11.16_evm.model.CTG_11.17

MALLRVLVCLFAVAISLNESAHMLQKKSFVQLGCMGMFDRSKFARLDAVCEECYELYREPDIHALCRSNCFKNSYFSKCVDALLLTGEQEELDNSVRQLYGKRK

**Diuretic Hormone 31 (DH31)**

>evm.model.CTG_308.2

MNPGISFRIFASMAAILVCCVLTSEAQMPSRYKRSIVEIDNPKETLQVLGELAQRILEAEGISNEKRGLDFGLSRGFSGSQAAKHLMGMSAASFANGPGRKRRFVDESNQ

>evm.model.CTG_674.24

MSPYSAIVLFMCITAATLDCSWSYPSSSAAQFRSKRSVDSEASEDIFRVLMRMAQDIVDTDSPVHEKRGIDLGISRGYSGSQAAKHLMGLAAANFANGPGRRRRDTESQ

**Diuretic Hormone 44 (DH44)**

>evm.model.CTG_556.25

MARSCVFVLACALAALAVCRARVMQGRGLLLGDDPQPGAAQQLLRSWGVLLAAARNSEDGDDPSSSSSEEQQPPNREVSKRRKGPTLSVVSPLEVLRQRLRLDRAKERIRENQKQIVANEELLEKIGKRASSSGILLLDKP

**Dsk**

>evm.model.CTG_1137.20

MTMKHLCTALAFLATFCLVTAAHPPRGKLERLARLVIGNPPNNRHNSLALARSLDNYAALARRQNDADSTSVDIGTTDFEDEDDVAIGKRTFDDYGHMRFGRSSATSREKKYDDYGHMRFGK

**Ecdysis-triggering hormone (ETH)**

>evm.model.CTG_173.7

MEKNDEQSACNGDQHENDPADNLDEESAAKAKLTDDPSKVKFTQVQLEGFGDAKNGDAKVDIHIAQTTVGMGKEELMKYANEPFWVRLRMFLFLFFWIAWVAMLVGAVVIIILAPRCPPAPKLEWYQKSAMYQVEVKGFMDGDGNGVGDFEGIASKIDYFKEQNIEAIMLSDFYKKSAATNGILDHKQVDSSIGTLEDFEKMVDKLRENNIRLVIDFNPNHSSDEHPWFTKSVEEQDSYKDFYIWADSPQNSLPNNWLNVNGEPAWTWNNQRRQFYLHTFGEHMPDLNLRNPLVRQELSEIVKFWLDKGVDGFKVVAASHLIEDADLRDEPVATPSQPNKRQYKDINHIYTVDQPENAGLFAEWKQILMNISASSGIPKILMPELSGEWNTSVIYYGNETVPLTELPFKAEFAEVRADISGVELKNLIHIQERPAYVWPTLLVGSRSVRRLATRVGHDLVDAIHMVSIFAKGTPIMYNGDEMGMTDVPSAKGGELKLLMTTMPWIGSDSTSGGFTNSSNSSYPVHPEFTNINVMAQLKEKQSHLKVFSRLLALRGQPALNFGLQEFPVVTDDIFSLLRVRKGSPGYLVTVNLGRNSTTVDFTGKSAVLPEMARVEVRGSNIVDGPLAEGEHPKIALNHTHHAWSSVLLVKKTVVRSRHKKNIYIWQMEVSAKYIFLAICLIMVLAKETNGQFFTKTTKNIPKMGRSVKTLPNLVHRVARTLHFVIQLVEQHDIDGNGELDPEELMEVPFIQNAAKNFYESKDGSTPYLDGSENEQEQEYDENRRMEINEHLIM

**Eclosion hormone (EH)**

>evm.model.CTG_1292.11

MISIIILAVVLTLTPNSVFGRNAGLCIHNCAQCKQTFGPFFEGQRCAEWCIRTQGSTLPDCSELDSIADFLTKLE

>evm.model.CTG_283.22

MSVTLGTVLAFVFCCTVAYSTGYSDSQWTSKFDAAEPSAFNDVNWDPAEPIVLRRRWVPESSYTPQLDVLRNRRLLQKLRRQKQEYDQQEVFGEISLIIAPSSSISRHFIMKTCYLIISMLLLFSASECVLSASMRTCIINCGQCKKMYGDYFLGQRCAEECIKTGGYLVPDCNEPSSIMRYLSKLA

>evm.model.CTG_283.23

MVFLKHLLCILVLLAVFDANLGLNPTNVCLKNCAQCKLTFGSYFNGPKCAESCIKYRGLLMPDCNDVKTIHGFLNKNE

**EFLamide (EFLa)**

>evm.model.CTG_1268.15

MTSCRHFAWIQMPILAVLLFVIFQTTNSASNDAEDAFSSDWTRDKPDQRVSEFMGGPGKRVSEFLGGPGKRLSEFLGGPGKRYSEFLGGPGKRYSEFLGGPGKRYSEFLGGPGKKRSSYYDLQEESSDKR

**Elevenin (Ele)**

>evm.model.CTG_1396.9

MYYVPQIGMLVVLLASWILSCQGLKDPDCSKMVFHPKCRGISAKRSSRLPFIDETEDSYNSEDSLEDSYWKNIIDSGLMERIMKEAIYASNYKKHLKSYYDEEK

>evm.model.CTG_147.17

MYYVPQIGMLVVLLASWILSCQGLKDPDCSKMVFHPKCRGISAKRSSRLPFIDETEDSYNSEDSLEDSYWKNIIDSGLMERIMKEAIYASNYKKHPKSYYDEEK

>evm.model.CTG_343.2

MTKFAEQSLVVFCCITCFCLIAEAIDCRKFVFAPRCRGVSAKRSAKLSNEELRELGEMEDSWGVDWPARSSVSDDAIADSATFESRRKAILQKLLEKFNDYNLHDDEN

**FMRFamide (FMRFa)**

>evm.model.CTG_1051.1

MKPRPKTFTSCHCMKMGLLMRFLFLTFIGRAVAFAISITSPETTNATRASLTSLNSGTESNNSKSRTTRAVLPSESNIWPHISEGAYLEEDNDDDEIFMESTDEDVDKRNIPMMRFGRSGASVIPSVRFGRSDESVIPGIRFGRSSAIPAMRFGRSESSVIPSIRFGRSESSVIPSIRFGRADASMIPTVRFGRSQSSAIPSMRFGRSGSSAIPTMRFGRSSSSAIPSIRFGRSSSSAIPSMRFGRSSSSVIPSIRFGRSSESMIPLVRYGRTDNSMIPSPRFGRTFKNTQDSDEDNEVEEIDSIAEHEAQNSDTNESEIDSDETGLDFISRNSKRESNESLIPQIRYGRSALKAKINKEVKSQYFGLPNIRPGRSSKDYLNTPEETNEPYLTDVQGFLGLFGAFRRSDDAIMGDSQNLQDMELGKKVEDFLLDANAENPYKNMDKQSLLSSQDTLYVIPYPRPGKRQLKMYPSTQKNIENSKGIGFQSVSKVNKIADFLVPYPRPGKKQEGALPLHYAKSQKPASVLPYPRPGRSKEMLPYPRPGKSTASLPYPRPGRSSDDVGRMIPYPRYGKSKYSLDSQSEKRSDQDHESTYLLNPLITEKIGNLKKHNSQETNSNPIFMLPYPRPGRDI

>evm.model.CTG_1152.6

MFSQTVSQGQRKKAVDSTFIRFGRSSPDHPGRNERSIDDIPDPESLSQIPRESMEEDFKTGPQRFLLDHNGCRVRDSTELMRALMSDPKCIRAFQKAKKENVNDTFIRFGKREAPPEREFEEAR

>evm.model.CTG_33.21

MTSYFLAGILVLVVLIENHVHIAATSSGMGSDSSDTPSIPILASGKRQHNIMRFGKRPSSGHSLMHFGKRDDDGPAPVPVKGHSFLYFGKRSSDQDQPSSFWDYFQSGSRGDGSYKRGHSIMRFGKRGGGPSDHPPHSFIRFGREIEDENDEDFDKRSGHSMLYFGKRGTGNAGHSMMYFGKRGSGNAGHSMMYFGKRGNNGHSMMYFGKRDDGELEDEEDSMDKRAHAMIHFGKRDDLVEEDKRAAHSMIHFGKRGEDPRKSSHAMIHFGKRDFDDEDSDEEEIDGFLDPQETMKRQHNLLRFGKKSEGKGSHAMIHFGKRDDLDEDKRAAHSMIHFGKRSAEEGSTAGNVSDKVNRKKREVAPLMPLARYNEEEEEVPVSEESLKQMQGFYGLLDHQEDEWEPSYIEYAGQPDKKETAKNAFLRFG

>evm.model.CTG_408.33

MAVHVAVTSFYFCLLLAAYTSLAEESLTSSVHATSLFKDRVHSSPDHSSTSTSNSKNVETNLLSPRNRRDLDPNQIRQFYNNNKVSNRALQSAFRYNDEILRNLLQSQASYNRNPYNSARSEFFYNILRPLDRRTTRYNSWDDHSVMHFGKRSIPSDDPRTIQLNGIESIPSDGQAHVEDSEEKLNLEKRSIPSDESRSIHSNSEESVLPDNSKSMSTDSEIKQKNVVENGGVVKRSSSWSDHGVLHFGKREYDENKLENKDDIPNHQNSSPSVEDSRKEEDRLPDQLDLSDIDKRDNNPWHSIMSFGKRNSNDPWHSVMSFGKRDGNDPWHNVMSFGKRDSNDPWHSVMSFGKRDGNEPWHNVMSFGKRDSNDPWHSVMSFGKRDGNDPWHSVMSFGKRDSNDPWHSVMSFGKRDGNDPWHNVMSFGKRDSNDPWHNVMSFGKRDSNDPWHNVMSFGKRDSNDPWHSVMSFGKRGENDPWHNVMSFGKRDGNDPWHNVLSFGKRAEKVSKDISNNNKENMDFLEKGLKQKRSIRETSEPYENELVPEKRFDVFKDHNTLHFGKKSDPWDSHNTLHFGKKSYPWDSHNTLHFGKKSHEWNSHNTMHFGKKSDPWESHTMHFGKKSDPWESHTMHFGKKSDPWESHTMHFGKKSDPWDSHNTMHFGKRSDVVHDSLTKVQKRKVLKDTTEQPVNSNTAVLDNLKLNKRSAAETKDGDSIKGKNPNNQEKIEKYEIRVKRSVILKNVDAIPKNTELKNTMLDNQKDVAGKKENQTSIESSKNKKGTKPEPKSTEGKSIASNIGKRSVVATENTVSNYITSPQINEEKSIKEPNSDRDKVLHFGKRSIALDDFENSMASDNQWNNEEEQLTKRSENYWDLHNTMHFGKKSDPWDSHNTMHFGKKSDPWESHTMHFGKKSDPWDSHNTMHFGKKSDPWDSHNTMHFGKKSDPWESHTMHFGKKSDSWDSHNTMHFGKKSDPWDSHNTMHFGKKSDPWDSHNTMHFGKKSDPWDYHNTMHFGKRSDPWESHNTMHFGKRSEPWDSHNTLHFGKRSDPWDSHNTMHFGKRSDPWDSHNTMHFGKRSDPWDSHNTLHFGKKFELSGNQSSLNSDFSVGIANDEGASSFLTVPEMMYLNDLKNSQDIRLKWNEYLSQLLAKLREGTITDSEVQSLKHYLTSLLEKGYISPEEPAFRNERLLLEFLEQFTGETSNKIPQAEYEEEDAADEEEDPDSGMKAVNKRYWAPFQGPKSIVYPHAWFASQIRRSAPGIEGPRMFETRVAEKKEDPVNSFMHFGKR

**Gonadotropin releasing hormone-related peptide (GnRH)**

>evm.model.CTG_40.4

MSAASRCALFLVLCVLVSSVLLCEAGTIHSSLGTGETTRGWKPGGKRAVDCWQLHSEALFAILEVVQEEVQKISRC

**GPA2**

>evm.model.CTG_314.6

HSRSIRIPGCVVFDITTNACRGFCSSFSTPSPEWIVRSNHRQSVWSVGQCCNIMETEDVYVEVMCLGGMKELRLNILKCISGKDWGASAKTLKITYTALIRPLLEYASPIWMQATRTNLEKLEKIQRNAARIICGLRNTTPGNISEFEAGLIPLRNRWDLNLTKFSEKRVNLKGHRTGDLIKDWIPSYRIKKKSPMHSILQNEIPITCPNKLTGSPVFPPCHKRKELIINLSIGNSVRKQDELPCLLRVLALEKINHFGPEVIKVYTDGSKDTVPARP

>evm.model.CTG_466.9

VFLVDQKEMFLAISKQAFMIKIFCIVIVVLMEQSTANIWEKPGCFKVGHTRTIGIPGCVEFDITTNACRGYCVSYSVPSSEDSLRINPKQVVTSYGQCCNIMETEDIKVKVMCLDGPKALTFKSAVTCACYHCKKN

**GPB5**

>evm.model.CTG_314.4

MLAPCPDRLFYLLALTAVFVVCSHAARGRSSDSNSQESYNLKSVLLCLRREYTLKATRSDNLGRRCWDFIRATSCWGRCDSYEIPDWRFPYKISIHPVCMHDQKRLRRVRLRHCDPPDADDRLRNYEYYDAETCSCGVCNSSDTFCDWKGHMHHHQRVVVPVQLPKKTPQEEEEEIDA

>evm.model.CTG_466.10

MLLVQPIGILILIASVSLGKTIVVDPENTLECHRREFTFKAVQTDDNGFQCWDQVTAMACWGRCDSGEIGDWRFPYKRPFHPVCVHDNREVRTIILRNCHPAADLRIAEYEYYEAVSCSCIKCDSSTTSCQGIPTFDAHPRKEKFPSIVTKPGRILPTKRMQFI

**Insulin-like peptide (ILP)**

>evm.model.CTG_1028.5

MPARLMLLSAVLLVACMHFMLGAGQESRLQKRSSVRACGRSLSQLISFVCQGVYNEPVATKRNSQFMLPEIDWTSEGNSIPEIDDYIFPMIRTRRGVVDECCHRSCTFSTLRSYCGVIPNREEKSD

>evm.model.CTG_823.19

MAWSSFPSVRRDVSFKMWKLLLLVVWLAAVTSDASPVLDPVRSLVKRGDNIRVCGRMLVDTLNMICEGEYYDPSDEQSRLAIGKRSIAPVDSYHDWIALTLRGASGNQERSQPPLQRYQRGVVDECCRKSCTMSTLSSYCSR

>evm.model.CTG_823.6

MAFKYLCLLLAVLMCCASALPRHQLLSRSKRSMRLCGSMLVDALAAVCESDYYDPSGRMEMRKRNYEYSNTDNFPGQYDSSITEFNGFVDPQTALDFLGLQRMVPPRASRGVADECCQKSCTFNQLASYCGNRGKRFASGEKK

**KYMGLamide (KYMGLa)**

>evm.model.CTG_640.2

MSSLIFSSSLTCEFLPKMKKIKMPITKIFCCVLLICGLAAFTTESPANPEFAPNLFPGERWSYPFDGYQIDSTPTRHTFGVSVMSDVNKEQSTPLTSIPMSSEFSKTKTSEMKNNVKVGEPSEPYDSIAEEPVKRPSDGNTRGSTYDPTLIMMGLGKRINTFYPEQKIVDIHEHEFTSSPLAQKVYLRVRRSTGTGVGEQEGNRTSNARSSAKNEGIPPISFSKKETNVDRDFSLEEIEKEYLAPKQEGNEVSSTSFSKKIKRSATHSVLERVTKGQKDFPLQQKKNAEKFDIKSSERLKGSAMHSMLDKEASDFESTLLRKMENGDIDLKQEAIKSVPNGSGDKKPSEDNNAKRNKRSLHGNKEDERKVIENSKTQDPTTRDTVNKNNSNKFHIDTPFEDSDREKRTVRQSTKKFAYDPALKYMGLGKRQKFRRGFSSNYDPAFQYMGLGKRNVVAKILKRQSYDPAFQYMGLGKRNNDLGSRKRESYDPALRFMGLGKREYVSPKSKLYLPVRKISKRASYDPAFHFMGLGKRSFPKLNFKKASFDPAFQYMGLGKRDENDKKKSYDPAFQYMGLGKRDNGDIYDNDKKKSYDPAFKYMGLGKRDENDKKKSYDPAFQYMGLGKRDNVDSYENDKKKSYDPAFQYMGLGKRVTDTIKKSYDPAFQYMGLGKRDNVAIYETDKKKSYDPAFQYMGLGKRDNVDIYEADKKKSYDPAFKYMGLGKRKYAEIGSKRDSYDPAFKYMGLGKRINSVVNSKNGSNYSFNRLKWFVNNPEFIHKELESDKIGMTKKGYFDPAFKYMGIGKRTEMIRKSSFDPALKYMGLGKRNKNTNLVAYANKQNSSFPNTDIQNEESKRSSYNELALNYMGLGKSDYGIQGSSTRDSNVHYSKPISYAKANQRIKRSLNYDPALQYMGLGKRDNLLSDNSASSSFNQLYLLKPSSLDTSETSEHFKRNSYYDPALQYMGLGKRGSLKQGKYDPALNFIGLGKRKDELKVAAKMSQSKELDPLSTKFSIPLLNTNDPEFKDEIFSNRPSKTVFYESKRQKREIEREDIEPDQESDYDDYDDEYFDPEAYPKLHRIDNIDNPHGITIFNGNLTDKHNDFDLIFDYDESPVFADCEGSATPCNEETKENILYEGSPKKYGDKSKTNENGLPNGYPIGIEKQEMQLPDMETSLFSTTTKPLHMREELVPHRRPGTPFSYFSDPRKKRNLEEDIFPIVTRTTRQVKPSGKPYPFHSNLLKKRSQVNTLATISSDTDIEVKRSANSYHRKKGDLSQVQTPAIISSGIASRINRSDTSYAHNHRNKRGFSEFQKQDTLLKPRYKSPKSNFGDTNPTMLKKLSQNFEVGVPMVKARAQNYKVKRSIGEIDMNRPRYNPGWVFIGLGKRSQAGGKVVQDKLDSRMSLITKCYQLLRKVNDAFRGIAGPRLAEAEPFWMTNVQGKNMKAFQNFKK

**LWamide neuropeptides**

>evm.model.CTG_451.79

MLPNGFRLLIISTLYTFSFATNYVSYYVPSQVYYMRPGTSFKVGNQMPPHPEPDAVLINGNNPGGPGIFGQPGGGPGVFGQPGGGPGVFGPPGGGPGVFGQPGGGPGVFGQPGGPGFGQPGGPGVFGQPGGPGVYGQPGNPGIFGPPSRGPGVYGQPGVFGQPGVFGHPGRRAIFGPPNGGPGMFGPPGILGYPPPGPFRHMRPPQPEGFDQDSSTWRFNNGYGPHFPEQSFVPNTRGSSDRAHPGHRNSHREVSDSEPTAEMFPLIDNVP

**Natalisin (NTL)**

>evm.model.CTG_1364.61

MDLRSRGEKGWRRIVDVWMYLCLIGMQLSLPALAEEYSSYLQSTGENADLISSKQEAEDKRSSSFFPLRGKKSDLDSPEIPVRYPFVSNQQLDDKRSQFYPLRGKKIPTEMKRASYFMPMRGRKDDQEWSEEDSIDKRMSSFMPMRGRKDGEEDSADKRMSSFMPMRGRKDDDESVDADDSMEKRMSSFMPMRGRKDDYDSEEKRMSFMPMRGKKKSGPMMEGYNHFGKSGMYTKDIMNLTSSSSGSNSNSKDNAYGRDEVMDQKSVGSIGNSEQTLNHTTRRRSLPLLSSEDRGGLLKSQFEFPSVSRLSTDLLEKRQSNFIPIKYREGSVETSSDGFLENQENKNFKHGFQKEKTLFVPMTEHSLPNEEYTNIHPFSKVMKRRSKFMPMRGRRQKEKNGASETNIFSPFSGTLNQIPLDEQTIKELSLEFNLTGDNHTEVKIEDFVTDDKRSSSFMAMRGRRGKNYFPYPFQFPSGIGSSLMARHYGGKIHPMTSTWGNAAKSRKFDWDSVKRAFHATRGKRFSNDHSNEIEPEPVENADNNDLSKTPATSSMEKVYVRKGWRDKRSIVRPRIFLKSQKHYIALKRPMSFFATRGKRMTNLADEPRIP

**Neuropeptide F (NPF)**

>evm.model.CTG_1122.36

MTMHSVLVSYFLVSLSVTVLASMSNGGGEPGEIIAELPTQPIVFNTPDELRTYLQELDKYFAIVGRPRFGRTSGSGFPRNVYPYIRANSVPSDIF

**Neuropeptide-like precursor (NPLP)**

>evm.model.CTG_813.23

MKFATGIWLMYLILAMVSLCSSAEGDNVPDKRYVAALAKNGRLPKFSNTEKKSSQESLEVLPTEDQMYKVHDDFLLEYPSYAENSNAEDSTKTESKRNVASLARMGRLNAIRFGRFQSNLVSNKRIDSEESREDPVPEANEDLDETNCIPEENDVAVEANPNSHEKRYISALARSGRLPVWHTQRQRLWGTGKRYDNLPYWDSSSYVNDNGELQGDSGSTEVQKIKRCKKQFGNEMEKRNLAAMAKNGRLPFSDGKRNIAALARDGKLQYSGKRTVPYFANYGKLPLTKFEPQKRNIQALARNGRLVFNIDQGAKRNIQSMARNGKLLVNNDLSDKRNIQSIARNGNVVFKVDEEGKRNVQSMARNGKLPFSSDQDGKRNVQSMARNGKLQFNRDQFGKRNVQSMARNGKLPFNSDQDGKRNVQSMARNGKLPFNGDQFGKRNIQSMARLGKLPFNSDQDGKRNVKSMARNGKLLFNNDQGGKRNIQSMARNGKLTSFGLSNDGKRNVAALPSGDENLPTFVISEENKGVDTALSRTETSPYGIYPGTKRNIQAMARNGNLPFNSYEDEKRNVQAMARNGYLPLNGDQKRNVQAMARNGNLPFNNYADGKRNIQSMARNGNMPISSYQNGKRNIQAMARNGKLLYNSYPSYKRSEEDSESFELDDEFELEKRNLAALARNGKLPFRTQDKKA

**Orcokinin (OK)**

>evm.model.CTG_1337.38

MILYVSCFLLVVIESTILVSGQEGPRQNHARLARNDKSLKPTFNGADVIYALIRTRSLDQFGGSQLIRTLDPLTGGQITKSVDQLATDSALDNFKGGEVIAVLDRLTGGDILGSLETVEGMEVLEALEKSVHAHGLHQHPKRTLDTLTGMAFGMSKRFDSLSGSTFGLSKRDFDEIDNVGFTGFAKRAPGGRGRGSTLALAKKNFDEIDRAGFEGYEKKNFDEIDRAGFESFEKRGKK

**PGWX3GLamide (PGWX3GLa)**

>evm.model.CTG_149.25

MGNNLKQLVFFCQILQRITSHVTEHPFDELREMMKLDKSRSNAEDFNALHDLEGIFREDLMSDGRISNRNGKVSAIQKKMDFKPKYDPGWKFIGLGKRPRQSEMYGYENRYDSFGPNDSEYYDDVKVKNPLVAMSTLMNFEVLNAIRLGIAEAAAKDIKKGGGKQSHQFSGNSAENNRFSGRSLYMGKQNVMYDPAWMLTTLGK

>evm.model.CTG_149.28

MAYGHVTEHPFDELREMMKLDKSRSNVEDFNALHDLEGIFREDLMSDGPISNRNGKVSAIQKKMDFKPKYDPGWKFIGLGKRPRQSETYGYENHYDSFGPNDSEYYDDVKVKNPLVAMSTLMNFEVLNAIRLGIAEAAAKDIKKGGGKQSHQFSGNSAENNRFSGRSLYMGKQNVMYDPAWMLTTLGK

**Proctolin (Proc)**

>evm.model.CTG_453.33

MTAFRREVLALVFLTLLSLSWLGTARYLPTRGDGSRREQIKELLRAILDMAPEERDTGRSSFPYDFQGGSVAKRSLHESEGPVSFQRETE

**Pyrokinin (PK)**

>evm.model.CTG_1051.3

MIYVDETEEQDVSDIDLAFEPRNVQVKIGKDVREEYTLLDELGRGKFGIVYRCTELATGRKLAAKFVVTPRPDDRKDVEREVEIMRMLQQPRLLQLYDAFDDGRKEMCLILELIEGGELFERVISDDFILTEKACSIFVRQICEGVEYMHSKNVLHLDMKPENILCVTRTGNQIKIIDFGLARKYDPKKKLQVLFGTPEFVAPEVVNFDKVSYTTDMWSVGVICYVLLSGLSPFMGDNDMETMANVTKAEYDFNDESFDNISEVAKDFIAKLLIKDSTQRMTSHQSLRHPWLRKGEPKKDTKLNKKKLKRFVIRRRWQKAVNALLALIAMGATI

>evm.model.CTG_578.1

MSRSAKCPIILWCKFALVSVLLQVSLGLDISDDENHQALVQLGRIEKPGFWTFVEDSNSQANGVIPFARLGRKLFKLPQTLIPYPRTGRELFDTDIFDYDDSLMSDYSWHNLADGPMADFFMDGKSLIPKRAIAAFPPRFGRKKRSLSAEDEQMENEDSERAQDKDSRSPSRNWFNLGDFEADMFLRQDRKPKSKNSFVPRVGKRHFERGMDEIPENNKRAKIFQPRVGRIPSSMLSRMESDNPRGLFQVKSRGAFIPRVGRRAAFVPRMGKKAKFSPRVGRRSEDDDRE

>evm.model.CTG_732.35

MEGMTAKFTACVTGIPTPDVCWLKDGQPLEPSSRHKMDLESSGILRLIIRDVEPSDYGSYSVTVSNNHGSATSAAKLLPDSLDEKYMTPIGDQFIDFDKFKKTGIPVPLSEKPRIVRMDDTSLTLGWKPSVPNSPRVPVTYQLEMAKHPDGEWTPYKTGLKDTLCDVRDLRPGQDYKFQVRVENKHGVSDPSPYVTAHRSKIYQPPKPDDFKPKDYSLEHQPLPKLAAPPKFVRKEEDTMYGVKGQPVTIEFWVYGHPEPQVTWFKGDSQIGKDKYGFMQDRNGKLCVFIDCMTDDFVGTYTCVAVNDEGEAQMKIKLAIAEHPVFLERLDETTAMSRRSARMQCRVTGLPYPKVKWFRDWHPLYESERVKILWEEPDKCTLFVSNLITRDNALYSCTATNIAGTATTSAALNVEDSEDMFDYKTYCRPTPVRPRTKIFEDFYDIGDELGRGTQGITYHAVKRDTGDSYAAKTMHGKGKLKEFMKNELDIMNQLCHPKLVRIRDAFETKDTLTLLTDICGGGELLNNIIKRGGVSERDIANYIKQILEGLDYMHSRYVGHLSLTIGDVMVSRVNSDDVRFGDFGVAARLLPGKDYYSEYGHPEFVAPEIAKKEPATVIADMWSVGVLTYLLLSGISPFLGENDRETLTRVQQGKINFVEDAFRGVSDDAKDFMSKLLVFDQKGRMDVKAALQHRWIKYYLDKPSSSERLNNLDRLKDYQRRWRDWYNNASCRRYYRRRTLESCFHHPSKMIYPPGESFTPSSSPEPLELDRTHAKPSHFDDVTFRQKINREDIDFRSESSYQNGPDTYLLQLRDTDFPLRLRQYLRVGAKSSPTIAASLKEGHWGSDRLVIRERRKFVDVMDEEIDDERKGLTKTRVPRRLYHEIGTLGFAREQMEELKKEVWKDKGSREMEIGMAPYFREKIRNSAMKENDEVVFRCYAVGTPKVDYTWFRNDGILLESSRIVVTRLKDGRCELRINPTRAYDIGVYKCVARNIHGTACCRARLKLGSKPGRPEPPVMKGSSDTEIYITWAVPRDEGNCTTLGYTLEKRLSGEENWELISNNIEHEYFVVRNLLPSTFYQFRVKAFNKFGWGEPGFPTEPLTTKPEGANKVKVSPRRKYQQEFTERTPDYVLEETEIPEFDYEQESNPVPLTEGDALDLYRIVSEMDRGRFSVVLNVWVKESNTSSIAKVIQSSKDADGRQEYDVMKGLVHERVVQLLSASHHVDKTVLVMEKLSGVDVMSYLAMRHDYNEELVVTIIKQVLEAIQYLHFREICYLELQPDNVVMVNLRDSDIKLVDFGTARFVPKTGAKVQVEGTPEYLAPEVLKQEPVSSLTDVWCVGVLTYILLSGVSPFAGKDDTETRENVTFVRYHFDNLHADVTSEVTRFLLNIFKQCPIKRLTIDECLEHKWLAPSEYMIMKRESARFLPDNLAAFAKRFHAKKYEAMDPEMLTSLGMSSSRNEMKIEDF

>evm.model.CTG_813.39

MSIYVDETDPGEEPEPDFQPRTIVVKKGKDVKSEYNLNQELGRGKFGTVHRCTEKATGKVFAAKFITTPKQQDRLDVEREVEIMTILQHPRLLQLYDAFDDGKKSMTLILEIIEGGELFERVIDEDFVLTEKAVTIFMRQICEGVEYMHSKNILHLDMKPENVLCISRTGNKIKLIDFGLARKYDPKKKLKVLFGTPEFVAPEVVCFESIGYTTDMWSVGVICYVLLSGLSPFMGDSDMETMANVTRSEWDFEDESFDDISEDAKDFISKLLIKDQKKRLNASQALKHPWLRRDKKKQEQAIDKKKLKRFVIRRRWQKAVNALIALIRMGATI

>evm.model.CTG_884.1

IYVTAMNSFQKSSRKSLRACAPRFVSPLSWSDAGCHRTVECRVEGYPRPDIRWERSSRPVANSRRVHITRTGDRCVLQIRMPCSEEDCGTYTCVATNNIGSVRTKVTLPGSNSITMNGSSHSSPKSLPKEWQSRSRSVEHSPISTSECKTLSSLPRNRSESGPCLQSKVSVPKSRVATFTSHICINNIEGDRLEYTSKQTKEATMDPNIKKLSQSMDDCRKNRRGFLNRVKIPDIFTSSKNCKATESPAKSKSSKVHSSLGSKFCQSSASLKDSFKTPQNSKTKFNSPITNDSSTGLSCQRRGNDTSVNGNAKQFKSVKRKDVLNGSKSNCAINATGQSETDELEVQLTTRERIAAYLKRFESSTNSVRPKDSKVNRLPRRTAESFKTVVPKIEKKNSRDETLFGKFRRRISSVDGGKKTAGLFEKDEKVTAPKSNGISTEKHKESNVQSRFRWRTSNVDDRTNISKNAKTEIDSHEQPSNVINITNGIESRLLTKENENEDNVFRKFEGEHIQPNKNTCVESVASKDNRQGSLKCDPNYVNSSRDAAVLRNSSESFLDDEGNGIMQLQDIYGFVNTTSDKEELLEINVENDVKTKDPISYVDDGRSFLKCTNYVDDLGSFKSIDVNLMDEFSDFSSEFCSSEKPNGETENIEVDRISDQVLLPDEKVLESKENLIPSIHIEQSPEEISFNKLEEDPIQTIIDDGPKELIDEPLSTSHASYQKPCRGKIKYSAPLLEEECVALVSGRPATPDPDPQRALLLSPARIVRGPQSVTVLRGESLTLAVGFDGRPAPKVVWMKGGRVLREEVGGRLSILEGAEMSAVTVSDVTADDSGKYVASVENEGGGDSCFASVAVVGIPEPPGSEPTASEVTNHSLVASWYGSMYDGGSVVTGYQVEMCTLPDQKWHKVASSVNTSCTVLGLSKGQKYVFRVRAENRYGLSHPSKESKVIRLDDFLQESSSDEDADGKEYPTPIAIESGNSFTERFDLKEEVGKGRFGVVYRCVENSSGRARAAKVIRCVKAKDRQKVHQEIDIMGRLHHPKLMHVLAAFESGRNMIVVMEYISGGELFERVIADDFVLTERDCVLFMRQICSGVAHMHARNVIHFDLKPENILCKTRSSHKIKIIDFGLARVYDGDESLRVLFGTPEFVAPEIINYEPVGPGSDMWSVGVICYVLLSGLSPFMGDSDPETFSNITRGEMDFDDEAFDEISQEAKLFISQLLVKQVKKRLSAEECLKHPWLATGGAEQKRPLSTEKLKRFIIRRKWQKSGTAIRALGRMVSLSRSSLGSSCDSPLHSPTSSRSRSSLVSRSETADSNESDVFQER

**Relaxin-like**

>evm.model.CTG_1085.8

MVGLTVAADMDIDPVLENIFKARSDDDWRAVWHTEKHRKCYHELLIHMDWVCKKDVYAVKRKKRTTEPFIEEQQAHRFLGRRRKRGASRHSLVKRGIIDECCHGSEGCSWEEYAEYCPANSRHRL

**RYamide (RYa)**

>evm.model.CTG_1474.1

MTDISVLAHVKRPLAVAALTILCCSLLCETRAQQQFYPNGRYGRRSVLPPLAESTQDFRVAVSADDSMMCKFTGYADFYKCTIKEISQANQVLN

>evm.model.CTG_1474.2

MTGSSLVAHVKRPLAVAALTLLCCSLLCETRAQQFYPNGRYGRRSVISPLAESTQDFRVEISADDTMICKFTGFADYYRCTRKEISHADQVLN

>evm.model.CTG_422.1

MTGSSLVAHVKRPLAVAALTLLCCSLLCETRAQQFYPNGRYGRRSVISPLAESTQDFRVEISADDTMICKFTGFADYYRCTRKEISHADQVLN

>evm.model.CTG_879.3

MTGSSLVAHVKRPLAVAALTLLCCSLLCETRAQQFYPNGRYGRRSVISPLAESTQDFRVEISADDTMICKFTGFADYYRKEISHADQVLN

**Short neuropeptide F (sNPF)**

>evm.model.CTG_1084.3

MNSGSALRVCSLLLVVMLMAAEMTSAAPYNDYDNLRDLYELLIRNEAASGGSGGASSSYNSHQMERKGGRSPSLRLRFGRRADPLWHAEAPSDAN

**SNPF**

>evm.model.CTG_974.6

MKFWVILFRSTAFFLAMQASVSQVASREVDCYTSLEDRGNHEHKLQMRFRRQVSDIDVRREPKLRMRFGIQAPKVDGQRKPELQVRIRDEASKNDETRKPKLRMRFERQVAEVDGHHEPELQMRTTNEASADDETREPKLRLRWGRQVPEVAGSEPRMLFRRHIPEDNKNLELKLRMLSSRNIRNVDRYREPQLRMRFGRQVPEFDGYL

**Tachykinin (TK)**

>evm.model.CTG_150.5

LYEFRYIRVSSGPIHSHCKIHWRKKDKLAAMANCGSFLQFLFLLFCCKLSCSSPIDEDQRMFNKINANAFIDRLIHYTPEMRLMLNGFLRYQNIAKRPEDSSNRSKSPADDPLATILATGFLGSRGKKDDGDELPVFANGFPAGRG

**Trissin (Tris)**

>evm.model.CTG_804.8

MTPASVTMAHRSINETVGIYIFSMLMMLTMLPPAHVVSMACNACGPECANACGTAMFRACCFNYNKKRSAPTQNMEHVTADAVANLSEGLDASPWMNENSGDTAIRIRYPKPYLAYKWAQLLGLSDRSLNKQQSGGDFLY
